# Genetic structure shows the presence of small‐scale management units in a relict tree species

**DOI:** 10.1002/ece3.10500

**Published:** 2023-09-12

**Authors:** Yuan‐Yuan Li, Min‐Yan Cui, Xiao‐Wei Le, Jun Gong, Kai Jiang, Xin Tong, Qian Zhang, Jia‐Hui Li, Hong‐Yue Li, Ling Lu, Jie Zou, Rong Wang, Xiao‐Yong Chen

**Affiliations:** ^1^ Zhejiang Tiantong Forest Ecosystem National Observation and Research Station, Shanghai Key Lab for Urban Ecological Processes and Eco‐Restoration, School of Ecological and Environmental Sciences East China Normal University Shanghai China; ^2^ Institute of Eco‐Chongming (IEC) Shanghai China; ^3^ Eastern China Conservation Centre for Wild Endangered Plant Resources Shanghai Chenshan Botanical Garden Shanghai China; ^4^ State Key Laboratory of Systematic and Evolutionary Botany, Institute of Botany Chinese Academy of Sciences Beijing China; ^5^ Shanghai Engineering Research Center of Sustainable Plant Innovation Shanghai China; ^6^ Shanghai Institute of Pollution Control and Ecological Security Shanghai China

**Keywords:** chloroplast DNA, endangered species, historical demographic dynamics, management units, *Metasequoia glyptostroboides*, nDNA microsatellites

## Abstract

Identifying conservation units is crucial for the effective conservation of threatened species. Previous cases are almost exclusively based on large‐scale but coarse sampling for genetic structure analyses. Significant genetic structure can occur within a small range, and thus multiple conservation units may exist in narrowly distributed plants. However, small‐scale genetic structure is often overlooked in conservation planning especially for wind‐pollinated and wind‐dispersed trees, largely due to the absence of dense and elaborate sampling. In this study, we focused on a representative endangered relict plant, *Metasequoia glyptostroboides*. Using both nuclear microsatellites (nSSRs) and chloroplast DNA (cpDNA) fragments, we sampled across the narrow distribution range of this species and determined its conservation units by exploring its genetic structure and historical demography. cpDNA haplotypes were classified into two groups, but mixed in space, suggesting that the existent wild trees of *M. glyptostroboides* cannot be divided into different evolutionarily significant units. However, using nSSRs, we detected strong spatial genetic structure, with significant genetic differentiation and weak gene flow between the samples in the east of the species' distribution range and other samples. The divergence between the two nSSR groups was dated to the Last Glacial Maximum (*c*. 19.6 kya), suggesting that such spatial genetic structure has been maintained for a long term. Therefore, these two nSSR groups should be considered as different conservation units, that is, management units, to protect intergroup genetic variations, which is likely to be the outputs of local adaptation. Our findings highlight the necessity to reveal small‐scale genetic structure and population demography to improve the conservation strategies of evolutionary potential of endangered plants.

## Graphical abstract

nSSRs‐based genetic structure and population demography analysis show two spatially segregated genetic groups with limited intergroup gene flow. Therefore, two management units should be set up to protect intergroup genetic variations.

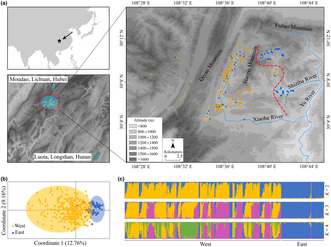

## 1 INTRODUCTION

One of the most important mechanisms causing the endangerment of species is insufficient intraspecific genetic diversity to keep pace with the ongoing environmental changes (Fricke et al., 2022). Therefore, protecting genetic variations and the genetic distinctiveness is essential for the conservation of threatened species (Liu et al., 2020; Meirmans & Hedrick, 2011; Teixeira & Huber, 2021). To achieve this goal, a key step in conservation practices is to divide the extant populations into different conservation units, including evolutionarily significant units (ESUs), which are developed to provide a rational basis for recognizing evolutionary heritage to prioritize protected populations, and management units (MUs), which are genetically differentiated units that can be monitored independently (Funk et al., 2012; Moritz, 1994). As significant genetic differentiation usually occurs over large regions, identification of conservation units was often performed at a broad scale (e.g., Olsen et al., 2014). However, populations can differentiate in a small range especially in mountainous regions due to local adaptation, founder effects by seeds stochastic colonization, and genetic drift in small and isolated populations (Frei et al., 2012; Steinfartz et al., 2007). Furthermore, given that distribution ranges of most threatened species are shrunken and that genetic differentiation within a small scale can be quickly formed in the presence of gene flow barriers (Vranckx et al., 2011), understanding the genetic structure to guide the design of small‐scale conservation units of endangered species is thus becoming increasingly urgent.

When identifying conservation units, multiple types of molecular markers are required to identify ESUs and MUs (Funk et al., 2012). To distinguish evolutionarily distinct taxa, ESUs should be reciprocal monophyly revealed by organelle DNA and show significant divergence of allele frequencies at nuclear loci (Moritz, 1994). For the convenience of conservation practices, groups of individuals with significant differentiations in allele frequency at nuclear or organelle DNA loci but not phylogenetic distinctiveness are also considered to be important, because they reflect intraspecific evolutionary potential and may represent functional differentiations, which is considered as MUs (Moritz, 1994; Olsen et al., 2014; Sandercock et al., 2022). Besides, another criterion of recognizing conservation units is that the observed strong genetic differentiations have been stable for the long term. However, this is seldomly tested in threatened species, mainly due to lack of knowledge about the demographic dynamics and the gene flow pattern.

Recently developed Bayesian approach has enabled the retrospection of historical demography using molecular markers and the inference of lineage split events and individual dispersal rates causing genetic differentiation (Beaumont, 2010; Setsuko et al., 2020), providing new insights into the delimitation of conservation units. In contrast to the traditional estimation of gene flow indirectly according to genetic differentiations, which has low statistical power to delineate gene flow pattern across different historical periods (Palsbøll et al., 2007), this approach can assess the strength of both historical and recent gene flow among differentiated groups and subsequently explore the role of historical events such as the Last Glacial Maximum (LGM). Therefore, the origin of genetic differentiation and spatiotemporal pattern of gene flow are available, which are crucial to test whether genetic differentiation is stable in the extant populations. Nevertheless, information of both genetic structure and demographic dynamics is still absent for most threatened species, largely because genetic factors are less considered than demographic characteristics when designing conservation strategies (Liu et al., 2020).

**FIGURE 1:**
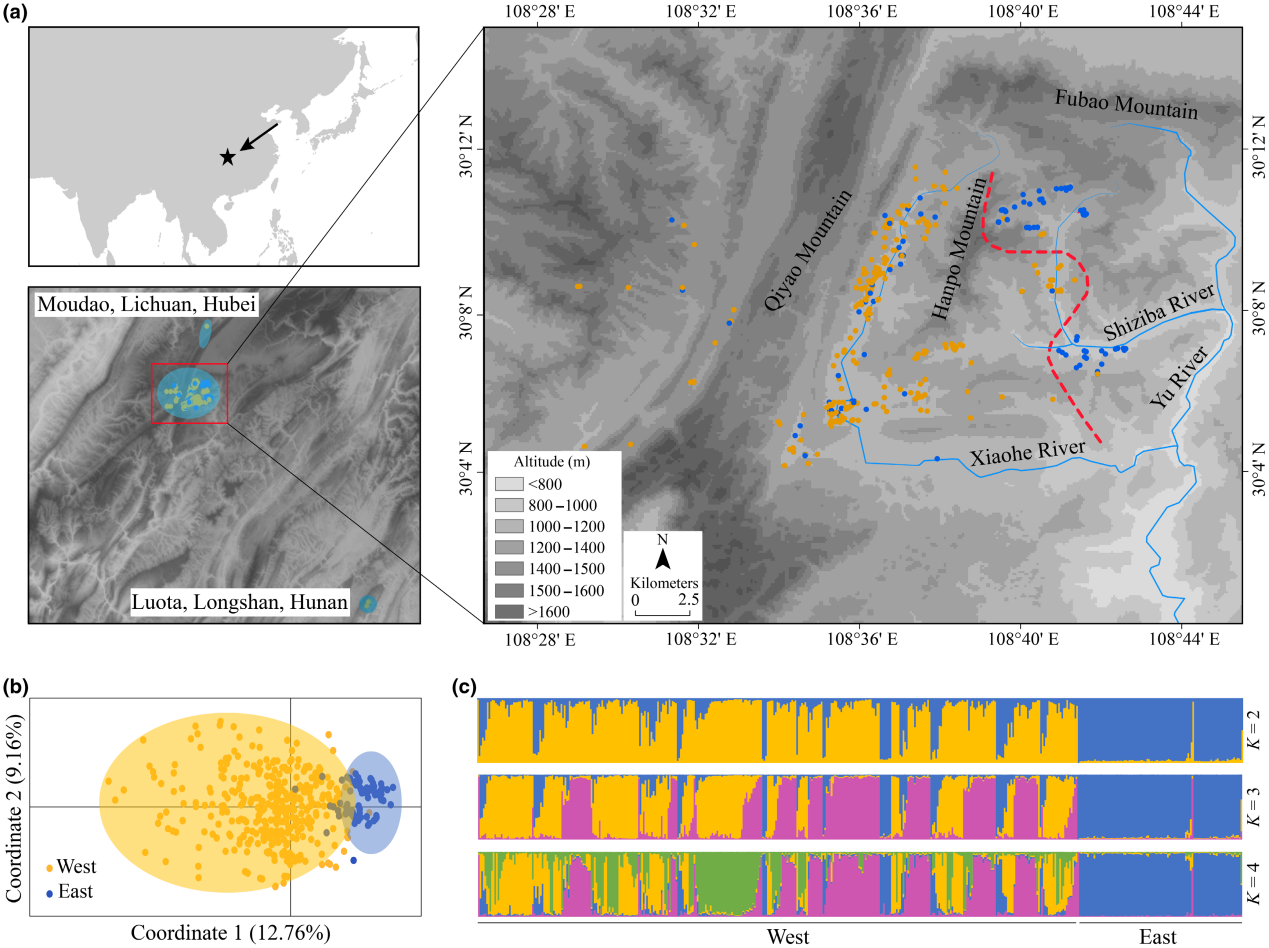
Sampling locations and genetic clustering of wild *Metasequoia glyptostroboides* trees using nSSR data. (a) The upper left figure shows the location of distributed area. The lower left figure shows the sampling region in the study. Blue backgrounds show the distribution range of the species. Red frame represents the region showed in the right figure, and dots at the north and south represent trees in Moudao town, Lichuan County, Hubei Province, and Luota town, Longshan County, Hunan Province, respectively. The right figure shows the main sampling area. Dots represent the locations of trees with different clusters by STRUCTURE (*K* = 2) using nSSR data, showing the topography and river network of the main distribution area. Blue lines represent the rivers along the mountainous valleys. Red dashed line indicates the division of the east and west groups according to the result of STRUCTURE. (b) Principal coordinates analysis (PCoA) based on nSSR genetic distances of individuals. The two genetic clusters inferred by STRUCTURE are represented by different colors. (c) Each individual is shown as a vertical line divided into segments representing the estimated membership proportions in the two, three, and four ancestral genetic clusters using STRUCTURE of nSSR data. Individuals within each cluster are arranged according to estimated cluster membership proportions.

*Metasequoia glyptostroboides* (Cupressaceae) is a well‐known “living fossil” tree species as well as a flagship species. It was thought to have been extinct in the early Pleistocene but was rediscovered in the 1940s in Three Gorges Mountain Region (TGMR), China. Fossil records indicate that genus *Metasequoia* originated in the early Late Cretaceous (*c*. 100 million years ago) and once distributed over most areas of the Northern Hemisphere (LePage et al., 2005; Li et al., 2012; Yang, 1998). The distribution range of this genus began to retreat during the Eocene and Miocene (Yang, 1998). At present, wild *M. glyptostroboides* trees can only be found in a mountainous region of the border area of Hubei Province, Hunan Province, and Chongqing Municipality (*c*. 800 km^2^), China, with a census size of about 5500 (Figure 1a). Because of its narrow distribution range and small population size, *M. glyptostroboides* was listed as an endangered species of IUCN (Farjon, 2013), and the first‐class of National Key Protected Wild Plants in China (http://www.forestry.gov.cn/). Our previous studies using random amplified polymorphic DNA markers detected a low genetic diversity in both its wild and planted populations (Chen et al., 2003; Li et al., 2005). Nevertheless, there are still no conservation measures concerning the protection of its genetic variations, largely because it is still unclear whether multiple conservation units exist within wild populations.

Complex landscape patterns often lead to population genetic structures through limiting gene flow and decreasing population size resulting in inbreeding and genetic drift (Love Stowell et al., 2017). Given the high complexity of landscape features in its natural distribution range, we hypothesized that strong genetic structure and multiple management units presented in this species. To test this hypothesis, 467 trees were sampled across its natural distribution range and were analyzed using both nuclear microsatellites (nSSRs) and chloroplast DNA markers. We analyzed the genetic structure and history demography. Specifically, we answered the two questions: (1) are there genetically differentiated groups of sampled trees in space; and (2) if yes, when did they form and was there recent gene flow after differentiation. We will delimit ESUs (by chloroplast DNA sequences) and MUs (by nSSRs) according to the definition proposed by Moritz (1994) and Palsbøll et al. (2007), to guide both in situ and ex situ conservation projects.

## 2 METHODS AND MATERIALS

### 2.1 Population information and tissue sampling

*Metasequoia glyptostroboides* is monoecious but mainly outcrossing. Both pollen and seed grains are dispersed by wind, with paternal inheritance of its chloroplast (Mogensen, 1996). Its typical habitats are humid forests and riparian zones. This species is distributed within a narrow and mountainous area with most wild trees (99% of wild trees) found in Lichuan County (Hubei Province, China), where the average annual rainfall is 1319 mm and the average temperature per month varies from 1.9 to 22.6°C (Li et al., 2021). In this area, *M. glyptostroboides* trees grow at elevations between 800 and 1400 m in two valleys (Xiaohe Valley in the west and Shiziba Valley in the east) separated by Hanpo mountain (the altitude of its peaks reaches 1400 m; Figure 1a; Tang et al., 2011). *M. glyptostroboides* is a dominant canopy species in local forests, and its adults can reach more than 30 m high with the diameters at breast height of adult trees of 30–350 cm (Li et al., 2021). In addition to Lichuan, scattered wild trees have been found in Shizhu (Chongqing Municipality) and Longshan (Hunan Province).

In this study, leaf tissues were collected from a total of 467 wild trees across its natural distribution range, including 443 trees from Lichuan, 21 trees from Shizhu, and three trees from Longshan (Figure 1a). The detailed sampling information is listed in Table S1. The collected fresh leaves were dried in silica gel for DNA extraction.

### 2.2 DNA extraction and PCR amplification

Total genomic DNA was extracted from sampled leaves using the revised CTAB method (Fan et al., 2004). A subset of samples (*n* = 172, Table S1) were amplified and sequenced using six fragments of chloroplast DNA (*trn*S‐*trnf*M (Shaw et al., 2005), *trn*C‐*pet*N2 (Lee & Wen, 2004), *rps*4‐*trn*S (Hennequin et al., 2003), *atp*H‐*atp*I (Grivet et al., 2001), *rpo*C1, and *psb*A‐*trn*H (Sass et al., 2007)). PCR amplification was performed in a Mastercycler (Eppendorf, Hamburg, Germany), with the process: 94°C for 5 min; 35 cycles of 94°C for 45 s, 50–55°C (annealing temperature for different primers) for 50 s, and 72°C for 90 s; and a final extension at 72°C for 8 min. The reaction mixture (50 μL) contained 5 μL 10 × Buffer, 2.5 mM Mg^2+^, 200 μM dNTPs, 0.2 μM primers, 5 U Taq DNA polymerase, and 50 ng template DNA. The PCR products were sequenced in both forward and reverse directions using an ABI 3730xl DNA Analyzer and edited using SeqMan Pro (DNASTAR). After testing for homogeneity among sequences using a partition‐homogeneity option implemented in PAUP* v.4.0b10 (Swofford, 2002), sequences of the six fragments were combined and then aligned using Clustal W (Thompson et al., 1994).

All of the 467 samples were genotyped using eight reciprocally independent microsatellite loci (Mg10, Mg 23, Mg37, Mg61, Mg64, Mg75, Mg76, and Mg77) developed for *M. glyptostroboides* by Cui et al. (2010). PCR amplifications were performed in 10 μL volume containing 50 ng template DNA, 0.1 mM of each primer, 0.2 mM of each dNTP, 1 × PCR buffer, 1.5 mM MgCl_2_, and 0.2 U of *Taq* polymerase. Amplification conditions were as follows: denaturation at 95°C for 5 min; 35 cycles of 94°C for 30 s, locus‐specific annealing temperature (53.5–65.0°C) for 45 s, and 72°C for 45 s; and a final extension at 72°C for 10 min. PCR products were scanned on an ABI 3130 genetic analyzer (Applied Biosystem, Foster City, CA, USA) using GeneScan 500(‐250) LIZ as the internal lane standard (Applied Biosystems). Amplified fragments were called and binned using GeneMapper 4.0 software (Applied Biosystems).

### 2.3 Genetic diversity and phylogenetic history based on cpDNA data

To estimate cpDNA diversity, we calculated number of haplotypes (*H*), haplotype diversity (*h*), and nucleotide diversity (*π*) using DNASP v. 6.12.01 (Rozas et al., 2017). Deviations from neutrality of sequence variations were tested by Tajima's *D* statistic and by Fu and Li's D* and F* statistics using DNASP v6.12.01.

To test whether there were multiple genetically differentiated groups within the sampled trees, a haplotype network was constructed using statistical parsimony as implemented in TCS v.1.2 (Clement et al., 2000). Ambiguities in the network were resolved using the criteria and procedures delineated by Crandall and Templeton (1993).

Furthermore, we constructed the phylogeny and estimated divergence time between clades constructing Bayesian phylogenetic trees using BEAST v.1.7.5 (Drummond et al., 2012). The sequences of *Cryptomeria japonica* (Taxodiaceae; GenBank accession number: AP009377), *Taiwania cryptomerioides* (Taxodiaceae; GenBank accession number: NC_016065), and *Cunninghamia lanceolata* (Taxodiaceae; GenBank accession number: NC_021437.1) were used as outgroups. The best model for nucleotide substitutions (the HKY + I + G model) was selected by the Akaike information criterion using jModeltest 2.1.4 (Darriba et al., 2012). Given that the phylogenetic tree was constructed for gymnosperms, a plant group with high probability of extinct species, a birth‐death speciation model with incomplete sampling was used as tree prior with estimated base frequencies (Stadler, 2010). Calibration points estimated from a molecular clock (Mao et al., 2012) were treated as priors on clade ages with a normal distribution. We used three molecular clock models (strict clock model, uncorrelated relaxed lognormal clock model, and uncorrelated relaxed exponential clock model). Three independent analyses were run for 30 million generations, sampling trees at every 3000 generations. Convergence to the posterior distributions of divergence times and parameter estimates was examined in TRACER v.1.5 (Rambaut & Drummond, 2007), and the burn‐in was set to be 10%. Based on Bayes factors calculated by TRACER, uncorrelated relaxed exponential clock was selected as the optimal clock model. Using this model, another two independent analyses were run. A maximum‐credibility tree was calculated using TREEANNOTATOR v.1.7.4 (Drummond et al., 2012) after removing trees sampled during the burn‐in phase.

To estimate the divergence time, we chose three molecular clock calibration points from Mao et al. (2012) based on the nucleotides of plastid, mitochondrial, and nuclear sequences. These calibration points were as follows: (1) the most recent common ancestor (MRCA) of Sequoioideae and Taxodioideae (139–215 million years ago (Mya)); (2) the MRCA of *T. cryptomerioides* and the common ancestor of *C. japonica* and *M. glyptostroboides* (157–241 Mya); and (3) the MRCA of *C. lanceolata* and other species of Taxodiaceae (157–260 Mya; Mao et al., 2012).

Genetic variation structure was estimated by analysis of molecular variance (AMOVA, Excoffier et al., 1992) on clade levels in GENALEX version 6.4 (Peakall & Smouse, 2006). Pairwise $\varphi_{\mathrm{ST}}^{\prime }$ was then estimated by *φ*_ST_/*φ*_ST(max)_ (Meirmans, 2006) between any two clades to infer the strengthen of pollen‐mediated gene flow, using the AMOVA approach in GENALEX.

### 2.4 Genetic structure and genetic diversity based on nuclear microsatellite data

Genetic diversity of eight SSR loci was estimated in GENALEX software using the following statistics: number of alleles (*N*_a_); number of effective alleles (*N*_e_); observed heterozygosity (*H*_O_); expected heterozygosity (*H*_E_; Nei, 1978); and inbreeding coefficient (*F*_IS_). We allocated all sampled individuals into 18 populations according to the locations of sampling sites to perform the linkage disequilibrium test and null allele frequency analysis (Table S2). Departure from Hardy–Weinberg equilibrium and linkage disequilibrium were examined using the exact tests in GENEPOP v.4.2.2 (Rousset, 2008) with sequential Bonferroni adjustment (Rice, 1989). The presence of null alleles was estimated using the software Micro‐Checker v.2.2.3 (van Oosterhout et al., 2004).

Bayesian clustering was applied to characterize population structure and estimate genetic admixture in all individuals using the software STRUCTURE version 2.3 (Pritchard et al., 2000). We ran 20 replicates of 10,000 Markov Chain Monte Carlo (MCMC) steps after 10,000 burn‐in steps for each of 1–15 genetic clusters (*K*). Admixture model and correlated allelic frequencies were used to analyze the data set. We used the Δ*K*, which measures the rate of change in the log probability of the data between successive *K* values (Evanno et al., 2005), to determine the optimal value of *K*. For the selected *K* value, clustering analysis was performed setting 1000,000 MCMC steps following 100,000 burn‐in steps. The output was visualized using the program DISTRUCT (Rosenberg, 2004).

Genetic diversity and differentiation between each pair of groups were estimated by Wright's *F*_ST_ using GENALEX, as it is an appropriate index when using molecular markers with high mutation rates such as microsatellites (Whitlock, 2011). Moreover, we also calculated $F_{\mathrm{ST}}^{\prime }$ (i.e., *F*_ST_/*F*_ST(max)_) to obtain standardized measures of genetic differentiation using AMOVA (Meirmans, 2006) in GENALEX. AMOVA was also performed to quantify genetic variations among groups, as implemented in GENALEX program. Principal coordinate analysis (PCoA) was used to visualize genetic divergence among samples in a multidimensional space based on distance matrix with data standardization, using GENALEX program.

To test the phylogeographic structure, we compared the global empirical $R_{\mathrm{ST}}$ and permuted $R_{\mathrm{ST}}$ ($pR_{\mathrm{ST}}$) using 10,000 permutations across allele size using the program SPAGeDi (Hardy & Vekemans, 2002). A significantly larger $R_{\mathrm{ST}}$ than $pR_{\mathrm{ST}}$ suggests that phylogenetically close alleles are more aggregated in a population than expected by random situations (Hardy et al., 2003; Hmeljevski et al., 2017).

Recent migration within the last several generations among each pair of groups was estimated using BayesAss v.3.0.4 (Wilson & Rannala, 2003). Markov chain Monte Carlo analysis was done with 1000,000 iterations, 100,000 burn‐in length, and 1000 samples. To examine the convergence, we used TRACER v.1.5 (Rambaut & Drummond, 2007) to analyze the trace file for the log probability of runs. We also estimate historical migration rates using Bayesian inference based on structured coalescent by Migrate‐n version 4.4.3 (Beerli et al., 2019). A Brownian motion model of mutation was used under Bayesian inference. We used three long chains 5,000,000 iterations in length and four static heating chains at temperatures 1.00, 1.50, 3.00, and 1000,000. The estimate of gene flow (*M*) was converted to the migration rate (*m*) divided by mutation rate (*μ*; where *μ* = 3.1 × 10^−4^ mutation per allele per generation; Hmeljevski et al., 2017; O'Connell & Ritland, 2004).

### 2.5 Demographic history estimation

The demographic history was also detected with an approximate Bayesian computation (ABC) procedure (Beaumont, 2010) following the Setsuko et al. (2020) and Jiang et al. (2021) approach. We estimated the population dynamics, population divergence, and historical migration based on all sampled trees using nSSR loci.

In the analysis of population size change, we calculated the average and standard deviation of the number of alleles, heterozygosity, and allele size range using ARLSUMSTAT v.3.6 (Excoffier & Lischer, 2010). For each genetic group, we compared the following three models (1) the standard neutral model (SNM) assuming a constant population size (*N*) over time; (2) the population growth model (PGM) where the ancestral effective population size *N*_a_ is assumed to increase exponentially (experiencing *t* generations ago) with a growth rate of *G*, that is, *N*_a_ = *N* × exp (*G* × *t*), to reach its current effective size (*N*); and (3) the size reduction model (SRM) which describes a population with an ancestral effective population size *N*_b_ experienced population decline from instantaneously *t*_1_ generations ago to reach its current effective size (*N*, *N* < *N*_b_). Details for prior distribution of parameters for each model are shown in Table S3.

The simulations of three models for nSSR data were performed by fastsimcoal2 v.2.6.0.3 (Excoffier & Foll, 2011). We used the generalized stepwise mutation model (GSM), setting the mutation rate per generation (*μ*) as 5 × 10^−4^ with the prior distribution for each locus drawn randomly from gamma distribution with shape and rate parameters (Setsuko et al., 2020) and the geometric parameter (*P*_GSM_) ranging from 0 to 1 for eight independent loci. All priors were generated by R v.4.0.3 (R Core Team, 2018). The three population size change models were simulated 100,000 datasets and summary statistics were calculated using ARLSUMSTAT. We then compared these three models by the ABC random forest approach using the R package ABCRF v.1.7 (Pudlo et al., 2016), with the number of trees in random forest setting as 1000. The classification error and posterior probability for each model were calculated to determine the best model. The goodness of fit for each scenario and priors was measured by the discrepancy between 1000 simulated pseudo‐observed datasets and our experimental data.

**FIGURE 2:**
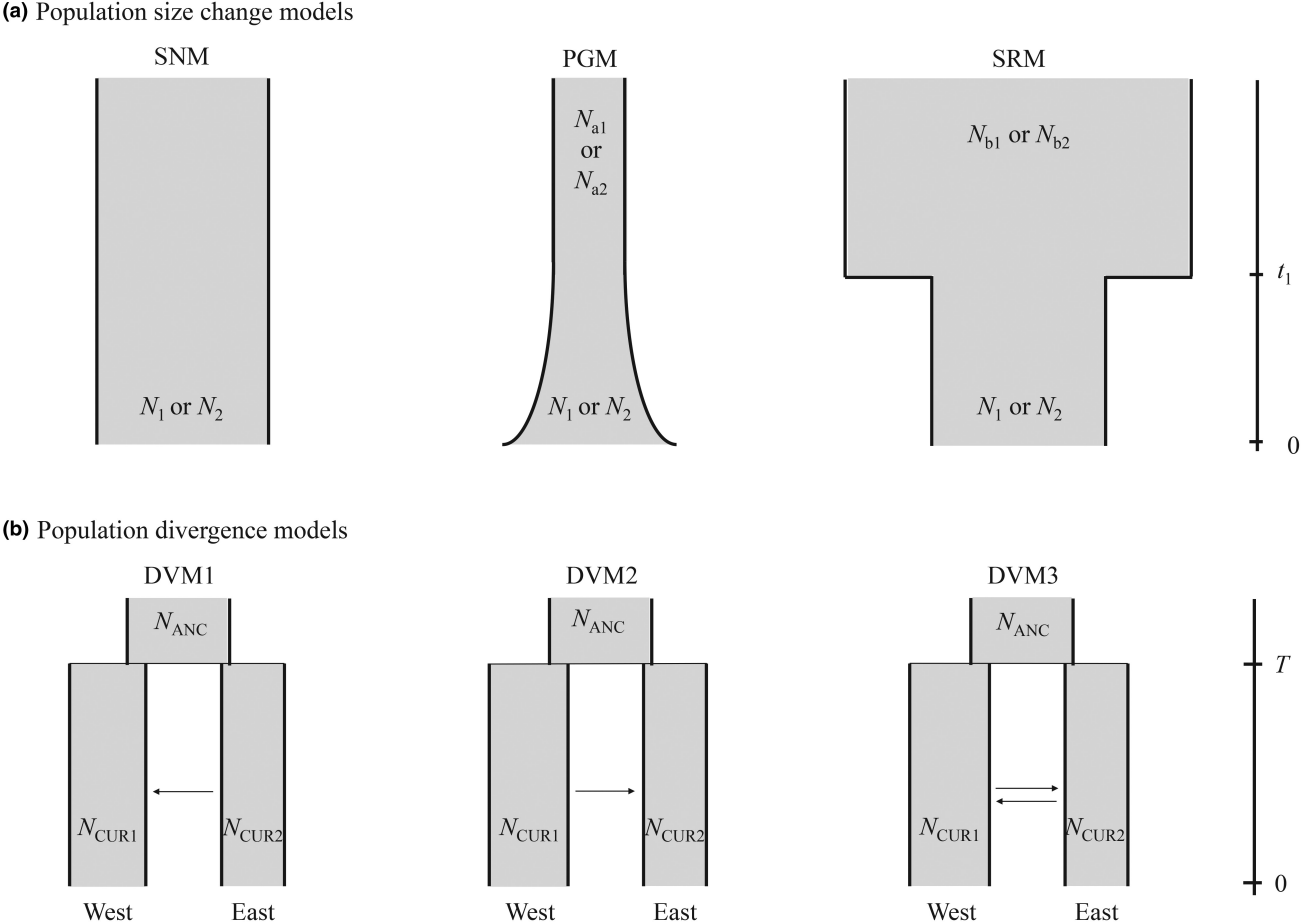
Demographic models compared in approximate Bayesian computation. (a) SNM (standard neutral model): the population size was constant at all the time. *N*_1_ and *N*_2_ were the current effective population sizes of the west and east groups, separately. PGM (population growth model): the population expanded at the time *T*. *N*_a1_ and *N*_a2_ were the effective population sizes of the west and east ancestral populations, respectively. SRM (size reduction model): the population size contracted at the time *t*_1_. *N*_b1_ and *N*_b2_ were the effective population sizes of the west and east ancestral populations, respectively. *t*_1_ = divergence time‐scaled by generation time; *N*_#_ = effective population size for two groups. (b) Divergence model (DVM) and dispersal model (DPM) at *T* generations ago with different gene flow. Arrows indicate the presence of gene flow.

Then three different population divergence models were compared (Figure 2). In population divergence analysis, the average total allele size range and *G*_ST(b)_ between the two clusters were calculated using ARLSUMSTAT, setting the other parameters using the same statistics of the best population dynamics model. The divergence model assumed the divergence between different genetic groups from their ancestral population with effective population size *N*_ANC_ at time *T*. Both unidirectional and bidirectional migration rates were set between any two genetic groups, forming three population divergence models. Each model has basic structural parameters: effective population size of a genetic group (such as *N*_CUR1_ and *N*_CUR2_), ancestral effective population size (*N*_ANC_, *N*_CUR_ < *N*_ANC_), divergence time (*T*), and migration rate (*m*). *N*_CUR1_ and *N*_CUR2_ were fixed according to the results in single population size model (Table S3). In all models, the period of migration was set from *T* to the present. Prior distribution of divergence time (*T*), ancestral effective population size (*N*_ANC_), and migration rate (*m*) were drawn from uniform distribution from 1 to 10^4^, from 10^2^ to 10^4^, and from 10^−5^ to 0.3, respectively. Simulations and model choice were conducted using the same approaches in the analysis of population dynamics. For the optimal population divergence model, we conducted 10^5^ simulations, with posterior distribution of parameters estimated using neural network regression by the R package ABC v.2.1 (Csilléry et al., 2012). The posterior mode and 95% highest posterior density were assessed using the density function and the R package coda (Plummer et al., 2006). The goodness of fit for the optimal model was evaluated using the difference between 1000 simulated pseudo‐observed datasets and our experimental data.

### 2.6 Delimitation of conservation units

Based on our results, we tried to delimit different types of conservation units for *M. glyptostroboides* using the following standards. Evolutionarily significant units (ESUs) were identified according to the definition from Moritz (1994), that is, within‐species lineages that were monophyletic for plastid DNA alleles with significant divergence of allele frequencies at nDNA loci. Management units (MUs) are different groups of populations with significant divergence of allele frequencies at nDNA or plastid DNA and of <10% of the dispersal rate of individuals between groups (Moritz, 1994; Palsbøll et al., 2007).

## 3 RESULTS

### 3.1 Genetic diversity and phylogenetic history based on cpDNA data

Based on the cpDNA data in 172 sampled trees, we identified a total of 47 distinct haplotypes (GenBank accession: OQ415011‐OQ415039), containing 17 variants including 10 SNPs and seven indels (Table S4). The overall haplotype diversity (*h*) and nucleotide diversity (*π*) were 0.926 and 0.90 × 10^−3^, respectively (Table 1). Results from both Tajima's test (*D* = 0.0596, *p* = .546) and Fu and Li's test (D* = 1.3996, *p* = .969; F* = 1.0674, *p* = .885) did not detect significant deviation from neutrality.

**TABLE 1:** Genetic diversity estimations sampled in 172 *Metasequoia glyptostroboides* trees in chloroplast DNA fragments.

Clade	Sample size	*H*	*h*	*π* (× 10^−3^)
Centered on haplotypes 1 and 7	84	17	0.684	0.39
Centered on haplotypes 9 and 10	88	30	0.931	0.91
Total	172	47	0.926	0.90

Abbreviations: *h*, haplotype diversity; *H*, haplotype number; *π*, nucleotide diversity.

**FIGURE 3:**
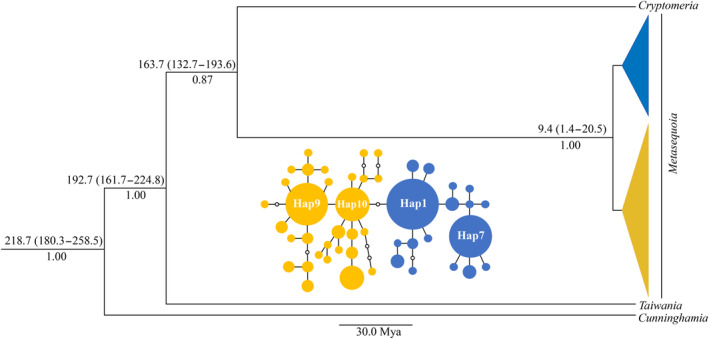
Chronogram for 47 haplotypes of *M. glyptostroboides* and three outgroups (*Cryptomeria japonica*, *Taiwania cryptomerioides*, and *Cunninghamia lanceolata*) based on chloroplast DNA fragments. Upper numbers represent divergent times (Mya, millions of years ago) with the 95% confidence interval of the mean time to the common ancestor in brackets, and lower numbers represent posterior probabilities. Phylogeny and divergent time are estimated through Bayesian methods in BEAST. Tip colors indicate the haplogroups as inferred by the phylogeny. Inserted small picture at the center indicates network of cpDNA haplotypes of *M. glyptostroboides*. The haplotype colors refer to the phylogeny analyses. The area of each circle is proportional to the frequency of each haplotype. Each solid line represents one mutational step that interconnects two haplotypes. The small hollow circles indicate ghost haplotypes.

After sequence alignment and removal of ambiguous regions, we obtained 2827 aligned nucleotides for all *M. glyptostroboides* haplotypes and three taxa (*C. japonica*, *T. cryptomerioides*, and *C. lanceolata*) for constructing phylogenetic trees. Using the Bayesian optimization approach, we found that all haplotypes of *M. glyptostroboides* were divided into two clades with a high posterior probability (1.00) (Figure 3), and the composition of haplotypes in each clade (centered on haplotypes 1 and 7, and centered on haplotypes 9 and 10, respectively) exactly matched with each group detected in the haplotype network (Figure 3). In addition, the phylogenetic relationships among haplotypes within each clade were poorly resolved (posterior probabilities <0.5 for all within‐clade nodes). These results showed the presence of two genetically differentiated clades in *M. glyptostroboides* based on cpDNA data. The divergence time between the two clades was estimated to be *c*. 9.4 Mya (95% HPD: 1.4–20.5 Mya) in Neogene (Figure 3).

Moderate genetic differentiation was found between the clades of sampled trees ($\varphi_{\mathrm{ST}}^{\prime }$ = 0.241), and AMOVA did not revealed a high intergroup differentiation (*F*_CT_ = 0.022, *p* = .003, Table S5). However, the sampled individuals assigned to the two genetic clades scattered in the study region and did not show a genetic structure in space (Figure S1).

### 3.2 Genetic diversity and genetic structure based on nuclear microsatellite data

Using the eight SSRs for all the 467 sampled trees, we detected 50 alleles in total, and *H*_O_ and *H*_E_ were 0.44 and 0.44, respectively (Table 2). The inbreeding coefficient per locus ranged from −0.648 to 0.541 with significantly high or low values in six loci, but the overall *F*_IS_ across all loci was only 0.003 (SE ± 0.041), showing a low level of inbreeding (Table 2). We did not detect significant linkage disequilibrium in any pair of loci. In addition, there was no solid evidence for the presence of null alleles (Table S6). We found a significant departure from Hardy–Weinberg equilibrium in loci Mg23, Mg61, and Mg76, due to a heterozygote deficit reflected by the positive *F*_IS_ values (Table 2). The genetic clustering analysis using STRUCTURE showed that when *K* = 2, most sampled trees located in different valleys were assigned into distinct genetic clusters (Figure 1c), forming the east and the west groups mainly segregated by the mountain lying between the two valleys (Figure 1a). Further division of sampled trees only occurred in the west group at *K* = 3 and 4 (Figure 1c). PCoA also clustered all sampled trees into an east and a west group, with the same assignment results as those in STRUCTURE analysis at *K* = 2 (Figure 1b). Therefore, these results supported the presence of two groups, despite that the optimal *K* was 3 according to the results of Δ*K* analysis (Figure S2). Note that the assignment of sampled trees based on nSSRs was inconsistent with the two cpDNA‐based genetic clades, which did not display apparent genetic structure in space.

**TABLE 2:** Genetic diversity calculated for eight loci and two groups from nuclear microsatellite data of 467 *Metasequoia glyptostroboides* sampled trees.

	*N*_a_	*N*_e_	*H*_O_	*H*_E_	*F*_IS_
Locus					
Mg10	4	2.092	0.546	0.499	−0.106*
Mg23	4	1.353	0.161	0.222	0.249*
Mg37	7	1.869	0.360	0.363	−0.006
Mg61	15	3.876	0.310	0.638	0.541*
Mg64	10	3.936	0.736	0.731	−0.031
Mg75	4	1.748	0.420	0.374	−0.087*
Mg76	3	1.244	0.093	0.153	0.320*
Mg77	3	2.225	0.893	0.537	−0.648*
Total	50	2.293	0.440	0.440	0.003
Group					
West	6.125	2.785	0.434	0.528	0.192*
East	3.500	1.930	0.440	0.359	−0.203*
Total	4.813	2.358	0.437	0.443	0.023

*Note*: Significant values are followed by *.

Abbreviations: *F*_IS_, inbreeding coefficient; *H*_E_, expected heterozygosity; *H*_O_, observed heterozygosity; *N*_a_, number of alleles; *N*_e_, number of effective alleles.

We found high genetic differentiation between the east and the west groups (*F*_ST_ (0.112) and $F_{\mathrm{ST}}^{\prime }$ (0.213)), and AMOVA also showed a significant genetic differentiation between the two groups explaining 18.34% of total genetic variance (Table S5). A phylogeographic structure was detected by a significant difference between the global estimates of $R_{\mathrm{ST}}$ and permuted $R_{\mathrm{ST}}$ ($R_{\mathrm{ST}}$ = 0.2268, ${\mathrm{pR}}_{\mathrm{ST}}$ = 0.0006, *p* < .001). Moreover, we detected weak but significant recent gene flow between the two groups, as of the lower boundaries of 95% confidence interval of migrate rates per generation (*m*) revealed by BayesAss were larger than 0, with the *m* from the west group to the east one being 0.0077 (95% CI: 0.0010–0.0208) and the *m* in the reverse direction being 0.0085 (95% CI: 0.0012–0.0192). A similar pattern of historical gene flow was also found using Migrate‐n, showing very low values of *m* (from the west to the east group: 0.0002 (95% CI: 0.0001–0.0003) and the reverse direction: 0.0004 (95% CI: 0.0002–0.0005)).

### 3.3 Demographic history and conservation units

Among the three population size change models, the standard neutral model (SNM) was the optimal model, which had the highest posterior probabilities for both groups. Average classification error rates of compared models were 0.295 and 0.315 of the west and east groups, respectively (Table S7). This result suggested that the population sizes of both groups were stable (Figure 2). In population divergence analysis, the optimal model was the divergence model with gene flow from the east group to the west one (DVM1) with a posterior probability of 0.502 (Table S7). In addition, for the optimal model in each analysis, most observed summary statistics were close to the simulated values based on parameter values generated from the posterior distributions (Figures S3 and S4), suggesting a high goodness of fitting.

Under these two selected models, the posterior mode values of effective population sizes were estimated to be 1327, 588, and 1975 for the west group (*N*_CUR1_), the east group (*N*_CUR2_), and their ancestral population (*N*_ANC_), respectively (Table 3). The posterior mode of divergence time *T* was assessed to be 783 generations ago (19,575 years ago with the generation length of 25 years; Table 3). Moreover, the posterior mode of the number of migrants per generation (*Nm*_21_) from the east group to the west one was very low (1.738), with a migration rate of 0.0013 (Table 3).

**TABLE 3:** Parameter estimates for the best demographic models based on approximate Bayesian computation of the west, east, and both groups.

Parameter	Mode	95% HPD	
		Lower	Upper
*N*_CUR1_	1327	940	1773
*N*_CUR2_	588	345	947
*N*_ANC_	1975	619	8971
*T*	783	27	5138
*Nm*_21_	1.738	0.014	59.715
*Shape*	1.594	0.534	4.335
*P*_GSM_	0.980	0.954	0.999

Abbreviations: mean *P*_GSM_, mean value of parameter in generalized stepwise nutation model among eight loci; *N*_ANC_, the ancestral effective population size; *N*_CUR1_, current effective population size of west group; *N*_CUR2_, effective population size of east group; *Nm*_21_, number of migrants per generation from east group to west one; *Shape*, a parameter of geometric distribution related to variance among eight loci; *T*, divergence time of west and east groups from their ancestral population.

Because there was no clear structure of the two cpDNA‐based clades, the wild *M. glyptostroboides* trees could only be considered as one ESU. However, using nSSRs, based on the high genetic differentiation of allele frequencies showed by STRUCTURE and PCoA (Figure 1), and weak gene flow detected by BayesAss and demography study between the two groups for nDNA loci, the wild *M. glyptostroboides* trees located in the east and west of the study area should be considered as two MUs.

## 4 DISCUSSION

Identifying conservation units is of great importance for the conservation of threatened species (Moritz, 1994). We identified two MUs in wild *M. glyptostroboides* trees using nSSR data, which locate in the east and west of our study area, with significant genetic differentiation and weak gene flow. However, it cannot be divided into different ESUs based on cpDNA information. Given that genetic factors have not yet been considered in the conservation of this species (Li et al., 2005), the determination of conservation units will greatly improve the conservation strategy of *M. glyptostroboides*.

The phylogenetic tree showed two clades in *M. glyptostroboides*, but the haplotypes mixed in space, supporting the presence of only one ESU. *M. glyptostroboides* was widely distributed across the northern hemisphere in the Paleocene (Lepage et al., 2005), and began to contract in the late Miocene and Pliocene probably due to the cool temperate and arid climate developed (Lepage et al., 2005; Zhang & Wang, 2015). Fossil records indicate that *Metasequoia* distribution continued to contract, and the remnant populations in China and Japan were under the control of warm temperate and moisture climate, until disappeared from Japan and other locations in China except the TGMR during the early Pleistocene (Liu et al., 2007). Thus, those haplotypes from different regions were admixed during the contractions and survived in the sole refugium.

Spatial Bayesian methods can overestimate the number of discrete clusters when faced with isolation by distance (IBD) pattern (Frantz et al., 2009). We thought two discrete groups could be identified across *M. glyptostroboides* distribution based on nSSR data by STRUCTURE and PCA, despite three clusters indicated by STRUCTURE without observed IBD and obvious biological explanation (Frantz et al., 2009). The refugium population of *M. glyptostroboides* was estimated to be formed at the early Pleistocene (Liu et al., 2007). This is consistent with the factor that the TGMR was one of the most important refugia for several ancient “tertiary relics” throughout the Quaternary, such as *Ginkgo biloba* (Hu, 1980; Shen et al., 2005), probably because effects of glaciations were far weaker than those in Europe and North America (Hu, 1980). Thus, we can confirm that the genetic structure (happened during the LGM) was formed in the current refugium. Moreover, the formation of two groups may be associated with the mountain separating two valleys. During the last glaciation, the mountain ridge might not have been an appropriate habitat (probably due to cold and dry climate) for *M. glyptostroboides*, which might have retreated to the areas of lower elevations, creating a long‐term discontinuous distribution. Though *M. glyptostroboides* is a wind‐pollinated and ‐dispersed species, it is still very difficult to disperse its propagules upwards over the ridge without any stepping stone conspecific trees or habitats. This is supported by the low but significant recent and ancient migration rates between the two groups. Thus, the mountain ridge was highly likely to act as the gene flow barrier, leading to the significant, though small‐scale, differentiation in nuclear DNA. Nevertheless, the warming climate after LGM may assist the return of *M. glyptostroboides* to the high elevations of the mountains and weaken the effects of gene flow barrier. This is probably why a few sampled trees assigned into the dominant genetic cluster in the western valley appeared in the eastern valley.

Difference spatial genetic patterns were revealed using two types of genetic markers. This is probably because chloroplast DNA and nSSR markers are designed to explore the genetic variations generated at distinct time scales. Chloroplast DNA is paternal‐inherited in gymnosperms, of which genetic variations mainly result from accumulated mutations with little or no crossing over (McCauley, 1995), and thus are able to probe the impacts of historical events. However, nSSR markers are expected to detect the recently formed genetic structure, due to the far higher mutation rate in nSSR regions than chloroplast DNA and frequent recombination of nSSR loci (Petit et al., 2005).

Species with limited distribution ranges and small population sizes often attract extensive attention of conservation because they face high risk of extinction (Pimm et al., 2014). To preserve the adaptative potential of an ESU in the face of rapidly changing environmental pressures, the total genetic variation harbored by all wild *M. glyptostroboides* trees should be maintained integrally (Li et al., 2020). Nevertheless, conservation practices should be conducted independently in each MU to protect the evolutionary potential of this relict species (Teixeira & Huber, 2021), as well as to achieve pathogen and pest prevention (Olsen et al., 2014). Thus, human‐mediated migration between MUs should be avoided, leading to the question that whether the two isolated MUs can persist. Generally, an effective population size (EPS) of 500 is considered as a threshold for long‐term persistence of a threatened species (Liu et al., 2020). Given the EPS of either MUs are larger than 500, such isolation is not likely to detrimentally impact MUs in the genetic aspect. In each MU, identification of wild individuals (e.g., trees containing rare alleles) for conservation priority must be first carried out to determine the core germplasm resources of *M. glyptostroboides* (Lu et al., 2007). Then, we can reinforce gene flow across different areas within each MU by facilitating developments of seeds and seedlings (Li et al., 2021), to maintain high levels of genetic diversity and low risk of inbreeding depression. Moreover, human‐assisted regeneration strategy like weeding is also necessary to improve the survival and growth of *M. glyptostroboides* seedlings and saplings (Li et al., 2021), for ensuring the effects of reinforced gene flow and enhancing the effective population size.

As to the ex situ conservation of *M. glyptostroboides*, although numerous planted populations have been established around the world, all of them are suffering from extremely low genetic diversity (Li et al., 2005), threatening their persistence in the presence of rapid environmental changes and pests/pathogens (Li et al., 2012). Therefore, it is essential to elevate the genetic variation in planted populations by collecting seeds from multiple trees in the core germplasm resources. However, it is worth noting that germplasms from different MUs should be introduced into distinct planted populations rather than introducing mixed germplasms (Li et al., 2005). Moreover, migration from planted population to the wild population must be prevented though there were many plantations nearby the distribution area of wild trees, in case of the loss of genetic distinctiveness due to genetic admixture. Although our findings can substantially improve current conservation strategy, future studies are expected to test whether differentiations in adaptation to environment conditions exist between MUs for the determination of suitable habitats for ex situ conservation and the prediction of the fate of *M. glyptostroboides* under the ongoing global changes.

Nowadays, drastic decline in population sizes and deprival of habitats by global changes and human disturbances have been reported in an increasing number of species (Díaz et al., 2019; Pimm et al., 2014). However, insights into small‐scale genetic structure and demographic dynamics are almost overlooked for threatened species with small distribution ranges, leading to less effective conservation measures. Our study provides an example that conservation units divided by the locally originated genetic structure can even exist within a very small range, highlighting the necessity to conduct small‐scale research when evaluating the existing genetic diversity for threatened species.

## AUTHOR CONTRIBUTIONS

**Yuan‐Yuan Li:** Conceptualization (equal); data curation (equal); funding acquisition (lead); investigation (equal); resources (equal); visualization (lead); writing – original draft (lead). **Min‐Yan Cui:** Formal analysis (equal); investigation (equal). **Xiao‐Wei Le:** Formal analysis (equal); investigation (equal). **Jun Gong:** Formal analysis (equal); investigation (equal). **Kai Jiang:** Formal analysis (equal); writing – original draft (equal). **Xin Tong:** Formal analysis (equal); writing – original draft (equal). **Qian Zhang:** Formal analysis (equal); writing – original draft (equal). **Jia‐Hui Li:** Formal analysis (equal); investigation (equal). **Hong‐Yue Li:** Formal analysis (equal); investigation (equal). **Ling Lu:** Formal analysis (equal); investigation (equal). **Jie Zou:** Formal analysis (equal); writing – original draft (equal). **Rong Wang:** Conceptualization (equal); writing – review and editing (equal). **Xiao‐Yong Chen:** Conceptualization (equal); methodology (equal); resources (equal); writing – review and editing (equal).

## CONFLICT OF INTEREST STATEMENT

The authors declare that they have no conflict of interest.

## Supporting information


Data S1

## Data Availability

Sequences for the chloroplast DNA sequences reported in this study are available under GenBank accession numbers OQ415011‐OQ415039. Microsatellite data are available in the Dryad Digital Repository https://doi.org/10.5061/dryad.z08kprrhv.

## References

[ece310500-bib-0001] Beaumont, M. A. (2010). Approximate Bayesian computation in evolution and ecology. Annual Review of Ecology, Evolution, and Systematics, 41, 379–405. 10.1146/annurev-ecolsys-102209-144621

[ece310500-bib-0002] Beerli, P. , Mashayekhi, S. , Sadeghi, M. , Khodaei, M. , & Shaw, K. (2019). Population genetic inference with MIGRATE. Current Protocols in Bioinformatics, 68, e87. 31756024 10.1002/cpbi.87 PMC9286049

[ece310500-bib-0003] Chen, X.‐Y. , Li, Y.‐Y. , Wu, T.‐Y. , Zhang, X. , & Lu, H.‐P. (2003). Size‐class differences in genetic structure of *Metasequoia glyptostroboides* Hu et Cheng (Taxodiaceae) plantations in Shanghai. Silvae Genetica, 52, 107–109.

[ece310500-bib-0004] Clement, M. , Posada, D. , & Crandall, K. A. (2000). TCS: a computer program to estimate gene genealogies. Molecular Ecology, 9, 1657–1659. 10.1046/j.1365-294x.2000.01020.x 11050560

[ece310500-bib-0005] Crandall, K. A. , & Templeton, A. R. (1993). Empirical tests of some predictions from coalescent theory with applications to intraspecific phylogeny reconstruction. Genetics, 134, 959–969. 10.1093/genetics/134.3.959 8349118 PMC1205530

[ece310500-bib-0006] Csilléry, K. , Francois, O. , & Blum, M. G. B. (2012). abc: An R package for approximate Bayesian computation (ABC). Methods in Ecology and Evolution, 3, 475–479. 10.1111/j.2041-210X.2011.00179.x 20488578

[ece310500-bib-0007] Cui, M. Y. , Yu, S. , Liu, M. , & Li, Y. Y. (2010). Isolation and characterization of microsatellite markers in *Metasequoia glyptostroboides* (Taxodiaceae). Conservation Genetics Resources, 2, 19–21. 10.1007/s12686-009-9132-6

[ece310500-bib-0008] Darriba, D. , Taboada, G. L. , Doallo, R. , & Posada, D. (2012). jModelTest 2: More models, new heuristics and parallel computing. Nature Methods, 9, 772. 10.1038/nmeth.2109 PMC4594756 22847109

[ece310500-bib-0009] Díaz, S. , Settele, J. , Brondízio, E. S. , Ngo, H. T. , Agard, J. , Arneth, A. , Balvanera, P. , Brauman, K. A. , Butchart, S. H. M. , Chan, K. M. A. , Garibaldi, L. A. , Ichii, K. , Liu, J. , Subramanian, S. M. , Midgley, G. F. , Miloslavich, P. , Molnár, Z. , Obura, D. , Pfaff, A. , … Zayas, C. N. (2019). Pervasive human‐driven decline of life on earth points to the need for transformative change. Science, 366, eaax3100. 10.1126/science.aax3100 31831642

[ece310500-bib-0010] Drummond, A. J. , Suchard, M. A. , Xie, D. , & Rambaut, A. (2012). Bayesian phylogenetics with BEAUti and the BEAST 1.7. Molecular Biology and Evolution, 29, 1969–1973. 22367748 10.1093/molbev/mss075 PMC3408070

[ece310500-bib-0011] Evanno, G. , Regnaut, S. , & Goudet, J. (2005). Detecting the number of clusters of individuals using the software STRUCTURE: A simulation. Molecular Ecology, 14, 2611–2620. 10.1111/j.1365-294X.2005.02553.x 15969739

[ece310500-bib-0012] Excoffier, L. , & Foll, M. (2011). Fastsimcoal: A continuous‐time coalescent simulator of genomic diversity under arbitrarily complex evolutionary scenarios. Bioinformatics, 27, 1332–1334. 21398675 10.1093/bioinformatics/btr124

[ece310500-bib-0013] Excoffier, L. , & Lischer, H. E. L. (2010). Arlequin suite ver 3.5: A new series of programs to perform population genetics analyses under Linux and windows. Molecular Ecology Resources, 10, 564–567. 10.1111/j.1755-0998.2010.02847.x 21565059

[ece310500-bib-0014] Excoffier, L. , Smouse, P. E. , & Quattro, J. M. (1992). Analysis of molecular variance inferred from metric distance among DNA haplotypes: Application to human mitochondrial DNA restriction data. Genetics, 131, 479–491. 1644282 10.1093/genetics/131.2.479 PMC1205020

[ece310500-bib-0015] Fan, X. X. , Shen, L. , Zhang, X. , Chen, X. Y. , & Fu, C. X. (2004). Assessing genetic diversity of *Ginkgo biloba* L. (Ginkgoacese) populations from China by RAPD markers. Biochemical Genetics, 42, 269–278. 10.1023/B:BIGI.0000034431.15308.57 15487590

[ece310500-bib-0016] Farjon, A. (2013). *Metasequoia glyptostroboides*. The IUCN red list of threatened species 2013: e.T32317A2814244. 10.2305/IUCN.UK.2013-1.RLTS.T32317A2814244.en

[ece310500-bib-0017] Frantz, A. C. , Cellina, S. , Krier, A. , Schley, L. , & Burke, T. (2009). Using spatial Bayesian methods to determine the genetic structure of a continuously distributed population: Clusters or isolation by distance? Journal of Applied Ecology, 46, 493–505. 10.1111/j.1365-2664.2008.01606.x

[ece310500-bib-0018] Frei, E. S. , Scheepens, J. F. , & Stöcklin, J. (2012). High genetic differentiation in populations of the rare alpine plant species *Campanula thyrsoides* on a small mountain. Alpine Botany, 122, 23–34. 10.1007/s00035-012-0103-2

[ece310500-bib-0019] Fricke, E. C. , Hsieh, C. , Middleton, O. , Gorczynski, D. , Cappello, C. D. , Sanisidro, O. , Rowan, J. , Svenning, J.‐C. , & Beaudrot, L. (2022). Collapse of terrestrial mammal food webs since the late Pleistocene. Science, 377, 1008–1011. 10.1126/science.abn4012 36007038

[ece310500-bib-0020] Funk, W. C. , McKay, J. K. , Hohenlohe, P. A. , & Allendorf, F. W. (2012). Harnessing genomics for delineating conservation units. Trends in Ecology & Evolution, 27, 489–496. 22727017 10.1016/j.tree.2012.05.012 PMC4185076

[ece310500-bib-0021] Grivet, D. , Heinze, B. , Vendramin, G. G. , & Petit, R. J. (2001). Genome walking with consensus primers: Application to the large single copy region of chloroplast DNA. Molecular Ecology Notes, 1, 345–349. 10.1046/j.1471-8278.2001.00107.x

[ece310500-bib-0023] Hardy, O. J. , Charbonnel, N. , Fréville, H. , & Heuertz, M. (2003). Microsatellite allele sizes: A simple test to assess their significance on genetic differentiation. Genetics, 163, 1467–1482. 10.1093/genetics/163.4.1467 12702690 PMC1462522

[ece310500-bib-0022] Hardy, O. J. , & Vekemans, X. (2002). SPAGeDI: A versatile computer program to analyze spatial genetic structure at the individual or population levels. Molecular Ecology Notes, 2, 618–620. 10.1046/j.1471-8286.2002.00305.x

[ece310500-bib-0024] Hennequin, S. , Ebihara, A. , Ito, M. , Iwatsuki, K. , & Dubuisson, J. Y. (2003). Molecular systematics of the fern genus *Hymenophyllum* s.l. (Hymenophyllaceae) based on chloroplastic coding and noncoding regions. Molecular Phylogenetics and Evolution, 27, 283–301. 12695092 10.1016/s1055-7903(02)00404-9

[ece310500-bib-0025] Hmeljevski, K. V. , Nazareno, A. G. , Bueno, M. L. , dos Reis, M. S. , & Forzza, R. C. (2017). Do plant populations on distinct inselbergs talk to each other? A case study of genetic connectivity of a bromeliad species in an Ocbil landscape. Ecology and Evolution, 7, 4704–4716. 10.1002/ece3.3038 28690800 PMC5496560

[ece310500-bib-0026] Hu, S. Y. (1980). The *Metasequoia* flora and its phytogeographic significance. Journal of the Arnold Arboretum, 61, 41–94.

[ece310500-bib-0027] Jiang, K. , Tong, X. , Ding, Y.‐Q. , Wang, Z.‐W. , Miao, L.‐Y. , Xiao, Y.‐E. , Huang, W.‐C. , Hu, Y.‐H. , & Chen, X.‐Y. (2021). Shifting roles of the East China Sea in the phylogeography of red nanmu in East Asia. Journal of Biogeography, 48, 2486–2501.

[ece310500-bib-0028] Lee, C. , & Wen, J. (2004). Phylogeny of *Panax* using chloroplast trnC‐trnD intergenic region and the utility of trnC‐trnD in interspecific studies of plants. Molecular Phylogenetics and Evolution, 31, 894–903. 15120387 10.1016/j.ympev.2003.10.009

[ece310500-bib-0029] LePage, B. A. , Yang, H. , & Matsumoto, M. (2005). The evolution and biogeographic history of *Metasequoia* . In B. A. LePage , C. J. Williams , & H. Yang (Eds.), The geobiology and ecology of Metasequoia (pp. 3–114). Springer.

[ece310500-bib-0030] Li, Y. Y. , Chen, X. Y. , Zhang, X. , Wu, T. Y. , Lu, H. P. , & Cai, Y. W. (2005). Genetic differences between wild and artificial populations of *Metasequoia glyptostroboides*: Implications for species recovery. Conservation Biology, 19, 224–231. 10.1111/j.1523-1739.2005.00025.x

[ece310500-bib-0031] Li, Y. Y. , Liu, C. N. , Wang, R. , Luo, S. X. , Nong, S. Q. , Wang, J. W. , & Chen, X. Y. (2020). Applications of molecular markers in conserving endangered species. Biodiversity Science, 28, 367–375. 10.17520/biods.2019414

[ece310500-bib-0032] Li, Y.‐Y. , Liu, Y.‐P. , Gong, J. , Fan, S.‐H. , Shen, G.‐C. , Zhou, Y. , Fang, Q. , Tang, Q. , Yang, Y. , Wang, R. , & Chen, X.‐Y. (2021). Unraveling the roles of various ecological factors in seedling recruitment to facilitate plant regeneration. Forest Ecology and Management, 492, 119219.

[ece310500-bib-0033] Li, Y. Y. , Tsang, E. P. K. , Cui, M. Y. , & Chen, X. Y. (2012). Too early to call it success: An evaluation of the natural regeneration of the endangered *Metasequoia glyptostroboides* . Biological Conservation, 150, 1–4.

[ece310500-bib-0034] Liu, C. N. , Li, Y. Y. , Wang, R. , & Chen, X. Y. (2020). Genetic factors are less considered than demographic characters in delisting species. Biological Conservation, 251, 108791.

[ece310500-bib-0035] Liu, Y. J. , Arens, N. C. , & Li, C. S. (2007). Range change in *Metasequoia*: Relationship to palaeoclimate. Botanical Journal of the Linnean Society, 154, 115–127.

[ece310500-bib-0036] Love Stowell, S. M. , Pinzone, C. A. , & Martin, A. P. (2017). Overcoming barriers to active interventions for genetic diversity. Biodiversity and Conservation, 26, 1753–1765. 10.1007/s10531-017-1330-z

[ece310500-bib-0037] Lu, H. P. , Wagner, H. H. , & Chen, X. Y. (2007). A contribution diversity approach to evaluate species diversity. Basic and Applied Ecology, 8, 1–12.

[ece310500-bib-0038] Mao, K. , Milne, R. I. , Zhang, L. , Peng, Y. , Liu, J. , Thomas, P. , Mill, R. R. , & Renner, S. S. (2012). Distribution of living Cupressaceae reflects the breakup of Pangea. Proceedings of the National Academy of Sciences of the United States of America, 109, 7793–7798. 10.1073/pnas.1114319109 22550176 PMC3356613

[ece310500-bib-0039] McCauley, D. E. (1995). The use of chloroplast DNA polymorphism in studies of gene flow in plants. Trends in Ecology & Evolution, 10, 198–202. 10.1016/S0169-5347(00)89052-7 21237002

[ece310500-bib-0040] Meirmans, P. G. (2006). Using the AMOVA framework to estimate a standardized genetic differentiation measure. Evolution, 60, 2399–2402. 10.1111/j.0014-3820.2006.tb01874.x 17236430

[ece310500-bib-0041] Meirmans, P. G. , & Hedrick, P. W. (2011). Assessing population structure: *F* _ST_ and related measures. Molecular Ecology Resources, 11, 5–18. 10.1111/j.1755-0998.2010.02927.x 21429096

[ece310500-bib-0042] Mogensen, H. L. (1996). The hows and whys of cytoplasmic inheritance in seed plants. American Journal of Botany, 83, 383–404. 10.1002/j.1537-2197.1996.tb12718.x

[ece310500-bib-0043] Moritz, C. (1994). Defining “evolutionarily significant units” for conservation. Trends in Ecology & Evolution, 9, 373–375. 21236896 10.1016/0169-5347(94)90057-4

[ece310500-bib-0044] Nei, M. (1978). Estimation of average heterozygosity and genetic distance from a small number of individuals. Genetics, 89, 583–590. 17248844 10.1093/genetics/89.3.583 PMC1213855

[ece310500-bib-0045] O'Connell, L. M. , & Ritland, K. (2004). Somatic mutations at microsatellite loci in western Redcedar (*Thuja plicata*: Cupressaceae). Journal of Heredity, 95, 172–176. 15073234 10.1093/jhered/esh024

[ece310500-bib-0046] Olsen, M. T. , Andersen, L. W. , Dietz, R. , Teilmann, J. , Harkonen, T. , & Siegismund, H. R. (2014). Integrating genetic data and population viability analyses for the identification of harbour seal (*Phoca vitulina*) populations and management units. Molecular Ecology, 23, 815–831. 10.1111/mec.12644 24382213

[ece310500-bib-0047] Palsbøll, P. J. , Bérubé, M. , & Allendorf, F. W. (2007). Identification of management units using population genetic data. Trends in Ecology & Evolution, 22, 11–16. 16982114 10.1016/j.tree.2006.09.003

[ece310500-bib-0048] Peakall, R. , & Smouse, P. E. (2006). GENALEX 6: Genetic analysis in excel. Population genetic software for teaching and research. Molecular Ecology Notes, 6, 288–295. 10.1111/j.1471-8286.2005.01155.x PMC3463245 22820204

[ece310500-bib-0049] Petit, R. J. , Dumimil, J. , Fineschi, S. , Hampe, A. , Sslvini, D. , & Vendramin, G. G. (2005). Comparative organization of chloroplast, mitochondrial and nuclear diversity in plant populations. Molecular Ecology, 14, 689–701. 10.1111/j.1365-294X.2004.02410.x 15723661

[ece310500-bib-0050] Pimm, S. L. , Jenkins, C. N. , Abell, R. , Brooks, T. M. , Gittleman, J. L. , Joppa, L. N. , Raven, P. H. , Roberts, C. M. , & Sexton, J. O. (2014). The biodiversity of species and their rates of extinction, distribution, and protection. Science, 344, 1246752. 10.1126/science.1246752 24876501

[ece310500-bib-0051] Plummer, M. , Best, N. , Cowles, K. , & Vines, K. (2006). CODA: Convergence diagnosis and output analysis for MCMC. R News, 6, 7–11.

[ece310500-bib-0052] Pritchard, J. K. , Stephens, M. , & Donnelly, P. (2000). Inference of population structure using multilocus genotype data. Genetics, 155, 945–959. 10835412 10.1093/genetics/155.2.945 PMC1461096

[ece310500-bib-0053] Pudlo, P. , Marin, J. M. , Estoup, A. , Cornuet, J. M. , Gautier, M. , & Robert, C. P. (2016). Reliable ABC model choice via random forests. Bioinformatics, 32, 859–866. 26589278 10.1093/bioinformatics/btv684

[ece310500-bib-0054] R Core Team . (2018). An Introduction to R, notes on R: a programming environment for data analysis and graphics (Version 3.5.0) .

[ece310500-bib-0055] Rambaut, A. , & Drummond, A. J. (2007). Tracer v1.5 . http://beast.bio.ed.ac.uk/Tracer

[ece310500-bib-0056] Rice, W. R. (1989). Analyzing tables of statistical tests. Evolution, 43, 223–225. 10.1111/j.1558-5646.1989.tb04220.x 28568501

[ece310500-bib-0057] Rosenberg, N. A. (2004). DISTRUCT: A program for the graphical display of population structure. Molecular Ecology Notes, 4, 137–138. 10.1046/j.1471-8286.2003.00566.x

[ece310500-bib-0058] Rousset, F. (2008). GENEPOP'007: A complete reimplementation of the Genepop software for windows and Linux. Molecular Ecology Resources, 8, 103–106. 10.1111/j.1471-8286.2007.01931.x 21585727

[ece310500-bib-0059] Rozas, J. , Ferrer‐Mata, A. , Sanchez‐DelBarrio, J. C. , Guirao‐Rico, S. , Librado, P. , Ramos‐Onsins, S. E. , & Sanchez‐Gracia, A. (2017). DnaSP v6: DNA sequence polymorphism analysis of large data. Molecular Biology and Evolution, 34, 3299–3302. 29029172 10.1093/molbev/msx248

[ece310500-bib-0060] Sandercock, A. M. , Westbrook, J. W. , Zhang, Q. , Johnson, H. A. , Saielli, T. M. , Scrivani, J. A. , Fitzsimmons, S. F. , Collins, K. , Perkins, M. T. , Craddock, J. H. , Schmutz, J. , Grimwood, J. , & Holliday, J. A. (2022). Frozen in time: Rangewide genomic diversity, structure, and demographic history of relict American chestnut populations. Molecular Ecology, 31, 4640–4655. 10.1111/mec.16629 35880415

[ece310500-bib-0061] Sass, C. , Little, D. P. , Stevenson, D. W. , & Specht, C. D. (2007). DNA barcoding in the Cycadales: Testing the potential of proposed barcoding markers for species identification of cycads. PLoS One, 2, e1154. 10.1371/journal.pone.0001154 17987130 PMC2063462

[ece310500-bib-0062] Setsuko, S. , Sugai, K. , Tamaki, I. , Takayama, K. , Kato, H. , & Yoshimaru, H. (2020). Genetic diversity, structure, and demography of *Pandanus boninensis* (Pandanaceae) with sea drifted seeds, endemic to the Ogasawara Islands of Japan: Comparison between young and old islands. Molecular Ecology, 29, 1050–1068. 10.1111/mec.15383 32048374

[ece310500-bib-0063] Shaw, J. , Lickey, E. B. , Beck, J. T. , Farmer, S. B. , Liu, W. S. , Miller, J. , Siripun, K. C. , Winder, C. T. , Schilling, E. E. , & Small, R. L. (2005). The tortoise and the hare II: Relative utility of 21 noncoding chloroplast DNA sequences for phylogenetic analysis. American Journal of Botany, 92, 142–166. 10.3732/ajb.92.1.142 21652394

[ece310500-bib-0064] Shen, L. , Chen, X. Y. , Zhang, X. , Li, Y. Y. , Fu, C. X. , & Qiu, Y. X. (2005). Genetic variation of *Ginkgo biloba* L. (Ginkgoaceae) based on cpDNA PCR‐RFLPs: Inference of glacial refugia. Heredity, 94, 396–401. 15536482 10.1038/sj.hdy.6800616

[ece310500-bib-0065] Stadler, T. (2010). Sampling‐through‐time in birth‐death trees. Journal of Theoretical Biology, 267, 396–404. 20851708 10.1016/j.jtbi.2010.09.010

[ece310500-bib-0066] Steinfartz, S. , Weitere, M. , & Tautz, D. (2007). Tracing the first step to speciation: Ecological and genetic differentiation of a salamander population in a small forest. Molecular Ecology, 16, 4550–4561. 10.1111/j.1365-294X.2007.03490.x 17877714

[ece310500-bib-0067] Swofford, D. L. (2002). PAUP*: Phylogenetic analyses using parsimony (*and other methods), version 4. Sinauer Associates.

[ece310500-bib-0068] Tang, C. Q. , Yang, Y. , Ohsawa, M. , Monohara, A. , Hara, M. , Chen, S. , & Fan, S. (2011). Population structure of relict *Metasequoia glyptostroboides* and its habitat fragmentation and degradation in south‐Central China. Biological Conservation, 144, 279–289.

[ece310500-bib-0069] Teixeira, J. C. , & Huber, C. D. (2021). The inflated significance of neutral genetic diversity in conservation genetics. Proceedings of the National Academy of Sciences of the United States of America, 118, e2015096118. 33608481 10.1073/pnas.2015096118 PMC7958437

[ece310500-bib-0070] Thompson, J. D. , Higgins, D. G. , & Gibson, T. J. (1994). Clustal W: Improving the sensitivity of progressive multiple sequence alignment through sequence weighting, position‐specific gap penalties and weight matrix choice. Nucleic Acids Research, 22, 4673–4680. 7984417 10.1093/nar/22.22.4673 PMC308517

[ece310500-bib-0071] Van Oosterhout, C. , Hutchinson, W. F. , Wills, D. P. M. , & Shipley, P. (2004). MICRO‐CHECKER: Software for identifying and correcting genotyping errors in microsatellite data. Molecular Ecology Notes, 4, 535–538. 10.1111/j.1471-8286.2004.00684.x

[ece310500-bib-0072] Vranckx, G. , Jacquemyn, H. , Muys, B. , & Honnay, O. (2011). Meta‐analysis of susceptibility of woody plants to loss of genetic diversity through habitat fragmentation. Conservation Biology, 26, 228–237. 22044646 10.1111/j.1523-1739.2011.01778.x

[ece310500-bib-0073] Whitlock, M. C. (2011). *G*'_ST_ and *D* do not replace *F* _ST_ . Molecular Ecology, 20, 1083–1091. 10.1111/j.1365-294X.2010.04996.x 21375616

[ece310500-bib-0074] Wilson, G. A. , & Rannala, B. (2003). Bayesian inference of recent migration rates using multilocus genotypes. Genetics, 163, 1177–1191. https://academic.oup.com/genetics/article/163/3/1177/6052984 12663554 10.1093/genetics/163.3.1177 PMC1462502

[ece310500-bib-0075] Yang, H. (1998). From fossils to molecules: The *Metasequoia* tale continues. Arnoldia, 58(59), 60–71.

[ece310500-bib-0076] Zhang, Y. , & Wang, J. J. (2015). Historical distribution of *Metasequoia* referenced to Paleoclimate factors. Botanica Pacifica, 4, 87–94.

