# Supplementary material for: Genetic structure shows the presence of small‐scale management units in a relict tree species
Source: Ecol Evol. 2023 Sep 12;13(9):e10500. doi: 10.1002/ece3.10500 (PMC10495812; doi:10.1002/ece3.10500)

**Table S1** Wild *Metasequoia glyptostroboides* distributed sites sampled, and voucher information.

| Location | Samples for nDNA analysis | Samples for cpDNA analysis | Longitude & Latitude | Altitude (m) | Voucher |
| --- | --- | --- | --- | --- | --- |
| Lichuan County, Hubei Province | 443 | 159 | E108°38'20'', N30°07'03'' | 1188 | Mg 0001 |
| Shizhu County, Chongqing Municipality | 21 | 10 | E108°29'03'', N30°08'36'' | 1472 | Mg sz06 |
| Longshan County, Hunan Province | 3 | 3 | E109°29'18'', N29°11'47'' | 893 | Mg LT01 |
| Total | 467 | 172 |  |  |  |

Voucher specimens were deposited in East China Normal University (ECNU), Shanghai, China.

**Table S2** The 18 wild *Metasequoia glyptostroboides* distributed sites sampled.

| Sites | Samples for nDNA analysis | Samples for cpDNA analysis | Location | Longitude & Latitude | Altitude (m) |
| --- | --- | --- | --- | --- | --- |
| Moudao | 1 | 1 | Lichuan County, Hubei Province | E108°41'02'', N30°26'16'' | 1383 |
| Zhongling | 33 | 8 | Lichuan County, Hubei Province | E108°36'46'', N30°10'33'' | 1341 |
| Guihua | 35 | 13 | Lichuan County, Hubei Province | E108°37'15'', N30°09'44'' | 1152 |
| Daocheba | 30 | 12 | Lichuan County, Hubei Province | E108°36'38'', N30°09'07'' | 1128 |
| Yangjiayuanzi | 23 | 8 | Lichuan County, Hubei Province | E108°36'25'', N30°08'20'' | 1121 |
| Guihuayuanzi | 11 | 10 | Lichuan County, Hubei Province | E108°36'10'', N30°07'38'' | 1154 |
| Xingguo | 41 | 12 | Lichuan County, Hubei Province | E108°38'20'', N30°07'03'' | 1188 |
| Gongqiao | 16 | 6 | Lichuan County, Hubei Province | E108°35'38'', N30°07'02'' | 1160 |
| Youjiawan | 35 | 13 | Lichuan County, Hubei Province | E108°37'40'', N30°06'24'' | 1202 |
| Xiaoheba | 31 | 10 | Lichuan County, Hubei Province | E108°37'06'', N30°05'29'' | 1169 |
| Xiaohe | 40 | 16 | Lichuan County, Hubei Province | E108°35'50'', N30°05'17'' | 1098 |
| Shanmu | 24 | 10 | Lichuan County, Hubei Province | E108°34'39'', N30°04'25'' | 1219 |
| Fanshen | 28 | 12 | Lichuan County, Hubei Province | E108°41'21'', N30°11'20'' | 1354 |
| Chatai | 42 | 5 | Lichuan County, Hubei Province | E108°40'54'', N30°10'04'' | 1305 |
| Honghua | 23 | 12 | Lichuan County, Hubei Province | E108°40'01'', N30°08'28'' | 1035 |
| Shiziba | 30 | 11 | Lichuan County, Hubei Province | E108°41'21'', N30°07'14'' | 939 |
| Huangshui | 21 | 10 | Shizhu County, Chongqing Municipality | E108°29'03'', N30°08'36'' | 1472 |
| Luota | 3 | 3 | Longshan County, Hunan Province | E109°29'18'', N29°11'47'' | 893 |
| Total | 467 | 172 |  |  |  |

**Table S3** Prior distribution of parameters in approximate Bayesian computation.

| Analysis | Parameter | Minimum | Maximum | Distribution |
| --- | --- | --- | --- | --- |
| Population size change | *N*_1_ | 1 | 2 × 10^3^ | Uniform |
|  | *N*_2_ | 1 | 10^3^ | Uniform |
|  | *N*_b1_ | 1 | 10^4^ | Uniform |
|  | *N*_b2_ | 1 | 10^4^ | Uniform |
|  | *G* | -0.05 | 0 | Uniform |
|  | *t*_1_ | 1 | 10^4^ | Uniform |
|  | *Shape* | 0.5 | 5 | Uniform |
|  | *P*_GSM_ | 0 | 1 | Uniform |
|  |  |  |  |  |
| Population divergence | *N*_CUR1_ | 1327 |  | Fixed value |
|  | *N*_CUR2_ | 588 |  | Fixed value |
|  | *N*_ANC_ | 10^2^ | 10^4^ | Uniform |
|  | *T* | 1 | 10^4^ | Uniform |
|  | *m* | 10^-5^ | 0.3 | Uniform |
|  | *Shape* | 0.5 | 5 | Uniform |
|  | *P*_GSM_ | 0 | 1 | Uniform |

**Table S4** Number, frequency and variations in chloroplast DNA haplotypes. H represents the haplotype; N is the number of haplotypes; Freq. is the frequency of the haplotypes; “–” represents the indel and “•” represents the same bases with corresponding points of the first line.

|  |  |  | Alignment Point | | | | | | | | | | | | | | | | | | | | | |
| --- | --- | --- | --- | --- | --- | --- | --- | --- | --- | --- | --- | --- | --- | --- | --- | --- | --- | --- | --- | --- | --- | --- | --- | --- |
| H | N | Freq. | *trn*S-*trnf*M | |  | *trn*C-*pet*N | |  | *rps*4-*trn*S | | | | | |  | *atp*H-*atp*I | | | | |  | *rpo*C |  | *pab*A-*trn*H |
|  |  |  | 37-38 | 633 |  | 738 | 739 |  | 986 | 996 | 1195 | 1234 | 1235 | 1236 |  | 1481-1482 | 1483-1484 | 1485-1486 | 1539 | 1615 |  | 1755 |  | 2411 |
| 1 | 31 | 0.1802 | –– | A |  | C | C |  | A | G | G | T | T | – |  | TA | TA | –– | C | C |  | G |  | A |
| 2 | 2 | 0.0116 | –– | • |  | • | • |  | • | • | • | • | • | T |  | • | • | –– | • | • |  | • |  | • |
| 3 | 4 | 0.0233 | TA | • |  | • | • |  | • | • | • | • | – | – |  | • | –– | –– | • | • |  | • |  | • |
| 4 | 3 | 0.0174 | –– | • |  | • | • |  | • | • | • | • | • | – |  | • | • | –– | • | • |  | A |  | • |
| 5 | 2 | 0.0116 | –– | • |  | • | • |  | • | • | • | • | – | – |  | • | • | –– | • | • |  | • |  | • |
| 6 | 1 | 0.0058 | TA | • |  | • | • |  | • | • | • | • | – | – |  | • | • | –– | • | • |  | • |  | • |
| 7 | 25 | 0.1453 | –– | • |  | • | • |  | • | • | T | • | • | T |  | • | –– | –– | • | • |  | • |  | • |
| 8 | 4 | 0.0233 | –– | • |  | • | • |  | • | • | T | • | • | – |  | • | • | –– | • | • |  | • |  | • |
| 9 | 24 | 0.1395 | –– | • |  | • | • |  | • | • | T | • | – | – |  | • | • | –– | • | • |  | A |  | • |
| 10 | 15 | 0.0872 | –– | • |  | • | • |  | • | • | T | • | • | – |  | • | • | –– | • | • |  | A |  | • |
| 11 | 1 | 0.0058 | –– | • |  | • | • |  | • | • | T | • | • | – |  | • | • | –– | • | T |  | A |  | • |
| 12 | 1 | 0.0058 | –– | • |  | • | • |  | G | • | T | • | – | – |  | • | • | –– | • | • |  | A |  | G |
| 13 | 2 | 0.0116 | –– | • |  | T | T |  | • | A | T | • | – | – |  | • | • | –– | • | • |  | A |  | • |
| 14 | 4 | 0.0233 | –– | • |  | • | • |  | • | • | T | • | • | T |  | • | –– | –– | • | • |  | • |  | G |
| 15 | 3 | 0.0174 | –– | • |  | • | • |  | • | • | T | • | • | T |  | • | –– | –– | • | • |  | A |  | • |
| 16 | 7 | 0.0407 | –– | • |  | • | • |  | G | • | T | • | • | T |  | • | –– | –– | • | • |  | A |  | • |
| 17 | 1 | 0.0058 | –– | • |  | • | • |  | • | • | T | • | • | T |  | • | • | TA | • | • |  | • |  | • |
| 18 | 4 | 0.0233 | –– | • |  | • | • |  | • | • | T | • | • | – |  | • | • | TA | • | • |  | A |  | • |
| 19 | 1 | 0.0058 | TA | • |  | • | • |  | • | • | T | • | • | – |  | • | • | –– | • | • |  | A |  | • |
| 20 | 1 | 0.0058 | TA | • |  | • | • |  | • | • | • | • | • | – |  | • | • | –– | • | • |  | • |  | • |
| 21 | 1 | 0.0058 | –– | • |  | • | • |  | • | • | T | • | – | – |  | • | • | TA | • | • |  | A |  | • |
| 22 | 1 | 0.0058 | –– | • |  | • | • |  | • | • | T | • | • | – |  | • | –– | –– | • | • |  | • |  | • |
| 23 | 1 | 0.0058 | –– | • |  | • | • |  | • | • | T | • | – | – |  | • | • | –– | T | • |  | A |  | • |
| 24 | 1 | 0.0058 | TA | • |  | • | • |  | • | • | T | • | • | T |  | • | –– | –– | • | • |  | • |  | • |
| 25 | 2 | 0.0116 | –– | • |  | • | • |  | • | A | T | • | – | – |  | • | • | –– | • | • |  | A |  | • |
| 26 | 2 | 0.0116 | –– | • |  | • | • |  | • | • | T | • | • | T |  | • | • | –– | • | • |  | A |  | • |
| 27 | 1 | 0.0058 | –– | • |  | • | • |  | • | • | T | • | • | – |  | • | • | TA | • | • |  | • |  | • |
| 28 | 2 | 0.0116 | TA | • |  | T | T |  | • | A | T | • | – | – |  | • | • | –– | • | • |  | A |  | • |
| 29 | 3 | 0.0174 | –– | • |  | • | • |  | G | • | T | • | – | – |  | • | • | –– | • | • |  | A |  | • |
| 30 | 3 | 0.0174 | –– | • |  | T | T |  | G | A | T | • | – | – |  | • | • | –– | • | • |  | A |  | • |
| 31 | 1 | 0.0058 | –– | • |  | • | • |  | G | • | T | • | • | – |  | • | • | TA | • | • |  | A |  | • |
| 32 | 1 | 0.0058 | –– | • |  | • | • |  | • | • | T | • | • | T |  | • | • | –– | • | • |  | • |  | • |
| 33 | 1 | 0.0058 | –– | • |  | • | • |  | • | • | T | • | – | – |  | • | –– | –– | • | • |  | • |  | • |
| 34 | 1 | 0.0058 | –– | • |  | • | • |  | G | A | T | • | – | – |  | • | • | –– | • | • |  | A |  | • |
| 35 | 1 | 0.0058 | –– | • |  | • | • |  | G | • | T | – | – | – |  | • | • | –– | • | • |  | A |  | • |
| 36 | 1 | 0.0058 | –– | • |  | T | T |  | • | • | • | • | – | – |  | • | • | –– | • | • |  | • |  | • |
| 37 | 1 | 0.0058 | –– | • |  | • | • |  | G | • | T | • | – | – |  | • | • | –– | • | T |  | A |  | • |
| 38 | 1 | 0.0058 | –– | • |  | • | • |  | G | • | T | • | • | – |  | • | • | –– | • | • |  | A |  | • |
| 39 | 3 | 0.0174 | –– | • |  | • | • |  | • | • | T | • | – | – |  | • | • | –– | • | T |  | A |  | • |
| 40 | 1 | 0.0058 | –– | G |  | • | • |  | • | • | T | • | • | – |  | • | • | TA | • | • |  | A |  | • |
| 41 | 1 | 0.0058 | –– | • |  | • | • |  | • | • | T | • | – | – |  | –– | –– | –– | • | • |  | A |  | • |
| 42 | 1 | 0.0058 | TA | • |  | T | T |  | G | • | T | • | • | – |  | • | • | –– | • | • |  | A |  | • |
| 43 | 1 | 0.0058 | –– | G |  | • | • |  | G | • | • | • | • | – |  | • | • | TA | • | • |  | A |  | • |
| 44 | 1 | 0.0058 | –– | • |  | • | • |  | G | • | • | • | • | – |  | • | • | –– | T | • |  | A |  | • |
| 45 | 1 | 0.0058 | –– | G |  | • | • |  | G | • | T | • | • | – |  | • | • | TA | • | • |  | A |  | • |
| 46 | 1 | 0.0058 | –– | G |  | • | • |  | G | • | T | • | • | – |  | • | –– | –– | • | • |  | • |  | G |
| 47 | 1 | 0.0058 | –– | G |  | • | • |  | G | • | T | • | • | – |  | • | • | –– | • | • |  | • |  | • |

**Table S5** Analysis of molecular variance (AMOVA) in chloroplast data of two clades and nuclear data of two groups of wild *M. glyptostroboides* trees.

| Source of variation | df | SS | MS | Variance component | Total variation (%) | *Nm* |
| --- | --- | --- | --- | --- | --- | --- |
| cpDNA | | | | | | |
| Among clades | 1 | 1.344 | 1.344 | 0.010 | 2.23 | 21.884 |
| Within clades | 170 | 77.092 | 0.453 | 0.453 | 97.77 |  |
| Total | 171 | 78.436 |  | 0.464 | 100 |  |
| nDNA | | | | | | |
| Among groups | 1 | 160.010 | 160.010 | 0.992 | 18.34 | 1.113 |
| Within groups | 465 | 2036.640 | 4.418 | 4.418 | 81.66 |  |
| Total | 466 | 2196.650 |  | 5.410 | 100 |  |

**Table S6** Frequencies of null alleles. Bold values correspond to loci with presence of null alleles.

| Sites | Mg10 | Mg23 | Mg37 | Mg61 | Mg64 | Mg75 | Mg76 | Mg77 |
| --- | --- | --- | --- | --- | --- | --- | --- | --- |
| Moudao | - | - | - | - | - | - | - | - |
| Zhongling | 0.0090 | 0.1208 | 0.0786 | **0.1638** | 0.0530 | 0.0464 | 0.1163 | -0.4523 |
| Guihua | 0.0852 | -0.0144 | -0.0290 | **0.3404** | -0.0097 | -0.0319 | -0.0290 | -0.6909 |
| Daocheba | 0.1190 | -0.3675 | -0.0238 | **0.2893** | 0.0539 | -0.2761 | **0.3435** | -0.7757 |
| Yangjiayuanzi | -0.0539 | 0.1326 | 0.0910 | **0.2343** | -0.0815 | -0.1136 | 0.1190 | -0.4210 |
| Guihuayuanzi | -0.1937 | 0 | 0.1803 | **0.2772** | **0.2584** | 0.0492 | -0.0465 | -0.4924 |
| Xingguo | -0.0979 | **0.2493** | 0.0554 | **0.2774** | -0.0302 | -0.1057 | **0.2382** | -0.4145 |
| Gongqiao | -0.4120 | -0.1632 | -0.1539 | **0.4082** | -0.1052 | -0.0963 | 0 | -0.5585 |
| Youjiawan | -0.0068 | 0.0749 | -0.0648 | **0.2032** | **0.1704** | **0.2719** | **0.3564** | -0.5799 |
| Xiaoheba | -0.0217 | **0.2762** | 0.0321 | **0.4227** | -0.1878 | -0.2043 | -0.0163 | -0.3841 |
| Xiaohe | 0.0450 | 0.0485 | -0.1292 | **0.4261** | 0.0367 | -0.1827 | **0.1854** | -0.5529 |
| Shanmu | **0.1502** | -0.1794 | 0 | **0.4268** | -0.2300 | 0.0728 | 0 | -0.6563 |
| Fanshen | -0.3634 | 0 | 0 | -0.5858 | -0.0900 | -0.5867 | 0 | -0.2208 |
| Chatai | -0.0466 | 0 | 0.0552 | -0.4059 | 0.0576 | -0.5526 | 0 | -0.2132 |
| Honghua | 0.0454 | 0.1410 | 0 | 0.0133 | **0.1066** | **0.2108** | 0.1174 | -0.4966 |
| Shiziba | -0.2472 | 0 | -0.1027 | **0.1658** | 0.0841 | -0.5929 | 0 | -0.5401 |
| Huangshui | 0.1587 | 0.1574 | -0.1959 | **0.3450** | 0.0917 | 0.0212 | 0 | -0.4021 |
| Luota | - | - | - | - | - | - | - | - |

“-” represents insufficient data to perform analysis due to the small numbers of individuals in Moudao (1 individuals) and Luota (3 individuals).

**Table S7** Proportion of votes by random forest composed of 1 000 trees based on a trained dataset of 100 000 simulations, posterior probabilities of alternative demographic scenarios and classification error in the west and east groups.

|  | model | Population size change | | Population divergence |
| --- | --- | --- | --- | --- |
|  |  | West group | East group | Both groups |
| Proportion of votes | SNM | **0.533** | **0.494** |  |
|  | PGM | 0.037 | 0.199 |  |
|  | SRM | 0.430 | 0.307 |  |
|  | DVM1 |  |  | **0.482** |
|  | DVM2 |  |  | 0.151 |
|  | DVM3 |  |  | 0.367 |
| Posterior probability |  | 0.509 | 0.569 | 0.502 |
| classification error rate |  | 0.295 | 0.315 | 0.315 |

Best model selected by RF is shown in bold. SNM, standard neutral model; PGM, population growth model; SRM, size reduction model; DVM1, divergence model with gene flow from east to west group; DVM2, divergence model with gene flow from west to east group; DVM3, divergence model with bidirectional gene flow; DPM1, dispersal model with gene flow from east to west group; DPM2, dispersal model with gene flow from west to east group; DPM3, dispersal model with bidirectional gene flow.

**Figure S1** Sampling locations and genetic clades of wild *M. glyptostroboides* trees using cpDNA data. Dots represent the locations of trees with different clades divided by phylogenetic tree using cpDNA data, showing the topography and river network of the main distribution area. Blue lines represent the rivers along the mountainous valleys.


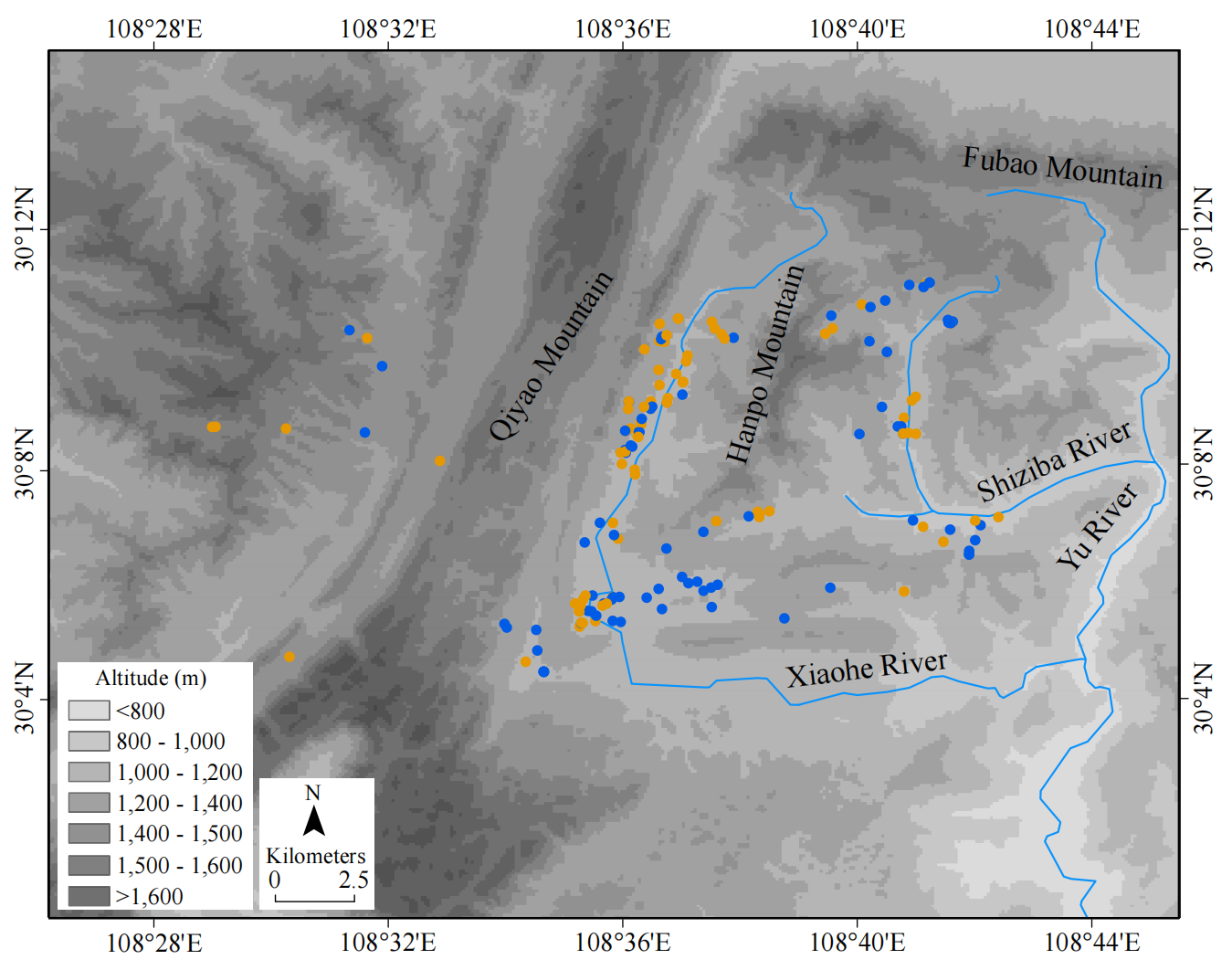


**Figure S2** Summary statistics in STRUCTURE including Δ*K* and Ln*P*(*K*).


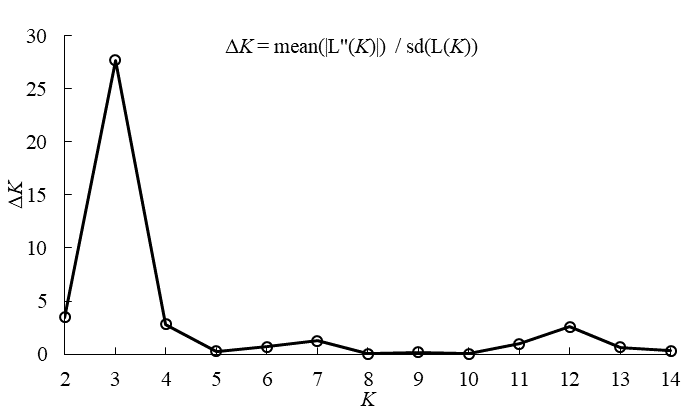

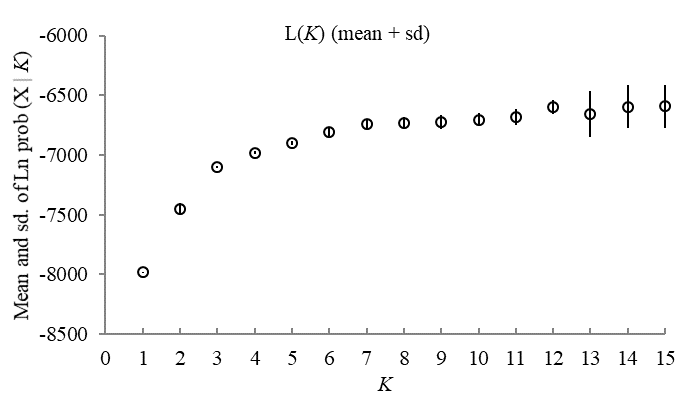


**Figure S3** Prior and posterior distributions of parameters for the best model in population divergence analysis, DVM2. Red ones are posterior with “neuralnet”, black ones are posterior with “rejection”, and dotted lines are priors.


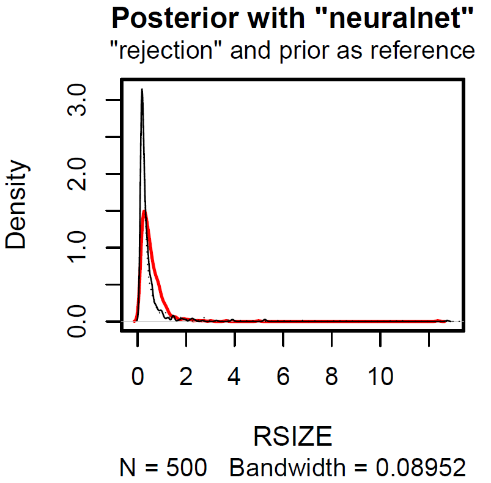

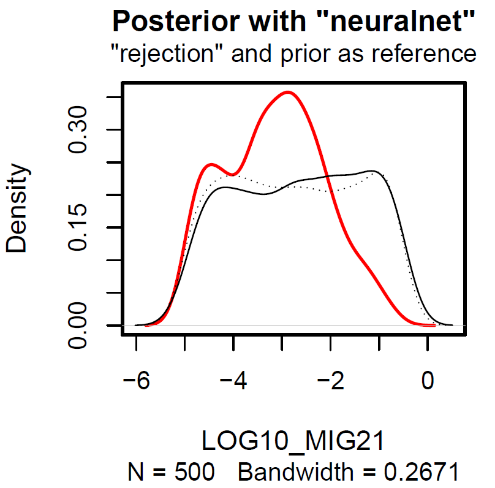

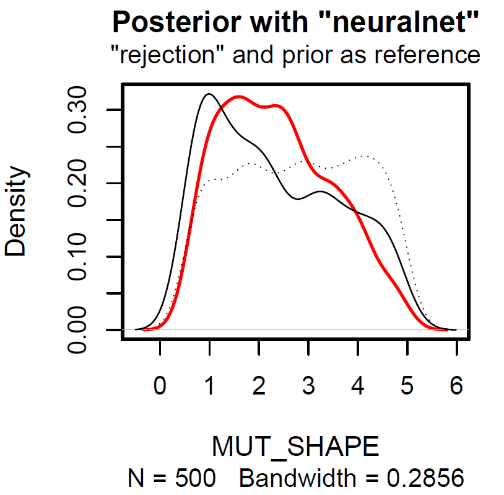

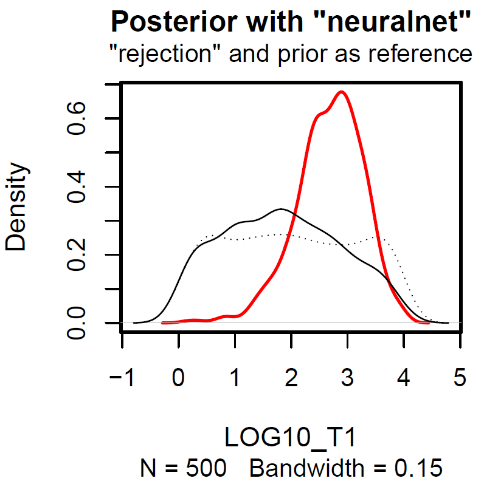

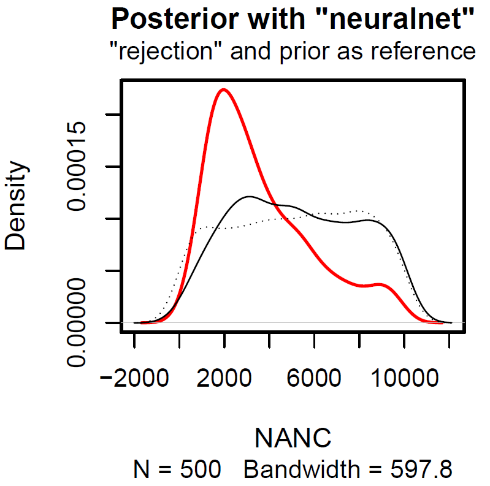

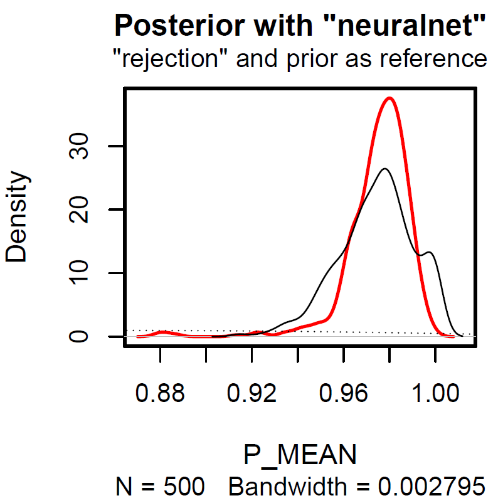


**Figure S4** Distributions of simulated and observed summary statistics in population divergence analysis. Simulations were conducted using 1,000 randomly drawn posterior of DVM2. Histogram and vertical bars indicate the simulated and observed values, respectively. K and Ksd are average and standard deviation of number of alleles among eight nSSR loci, respectively. H and Hsd are average and standard deviation of heterozygosity among eight nSSR loci, respectively. R and Rsd are average and standard deviation of allele size range among eight nSSR loci, respectively. 1 and 2 indicate the west and east group, respectively. FST is an average F_ST_ among eight nSSR loci.


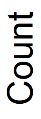

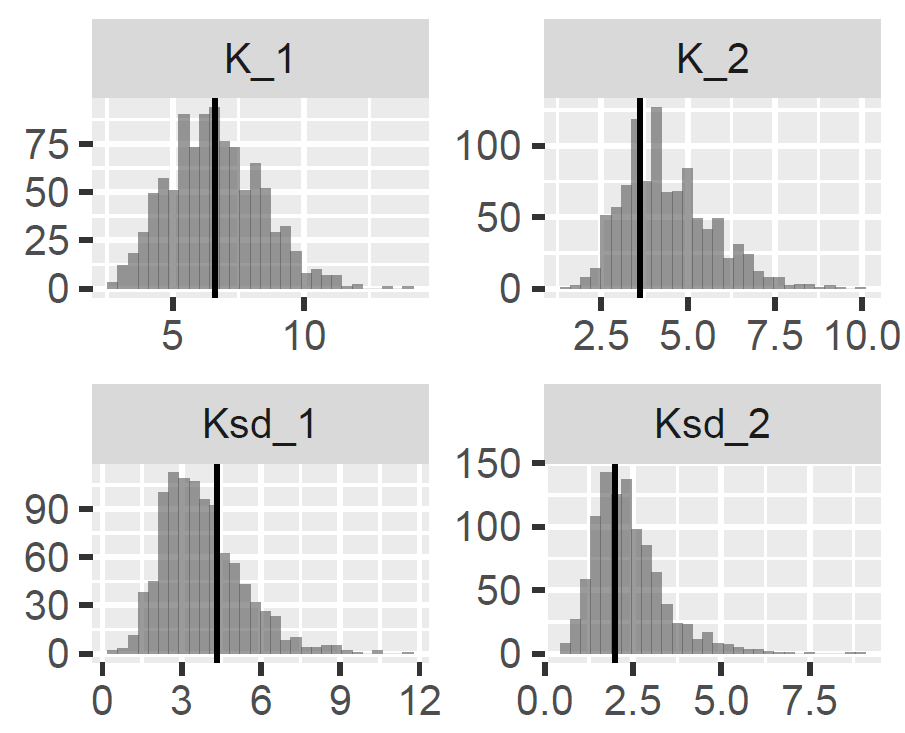

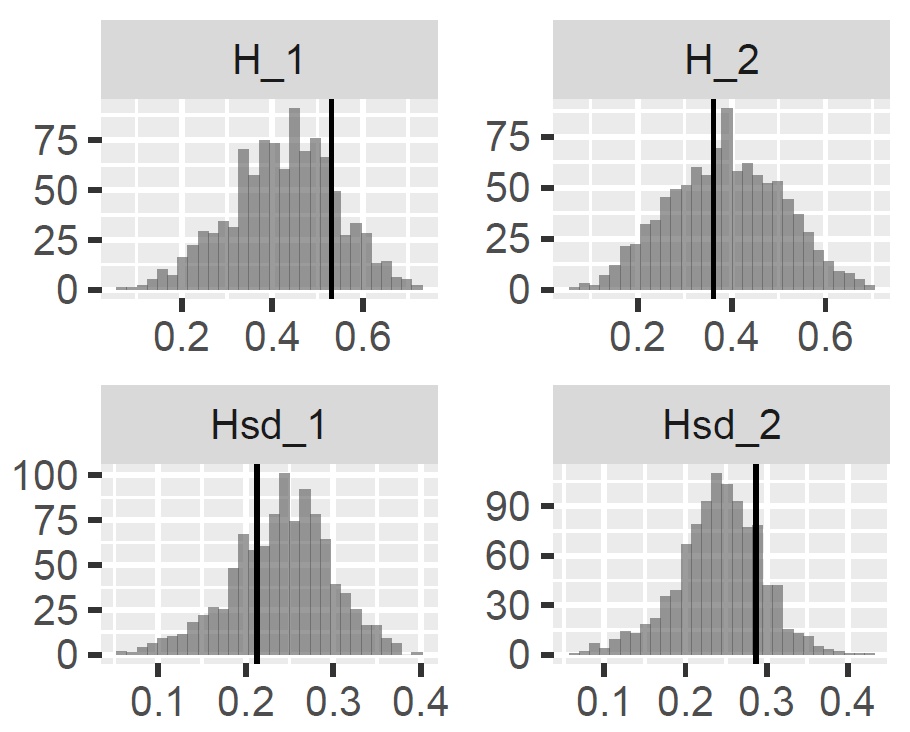

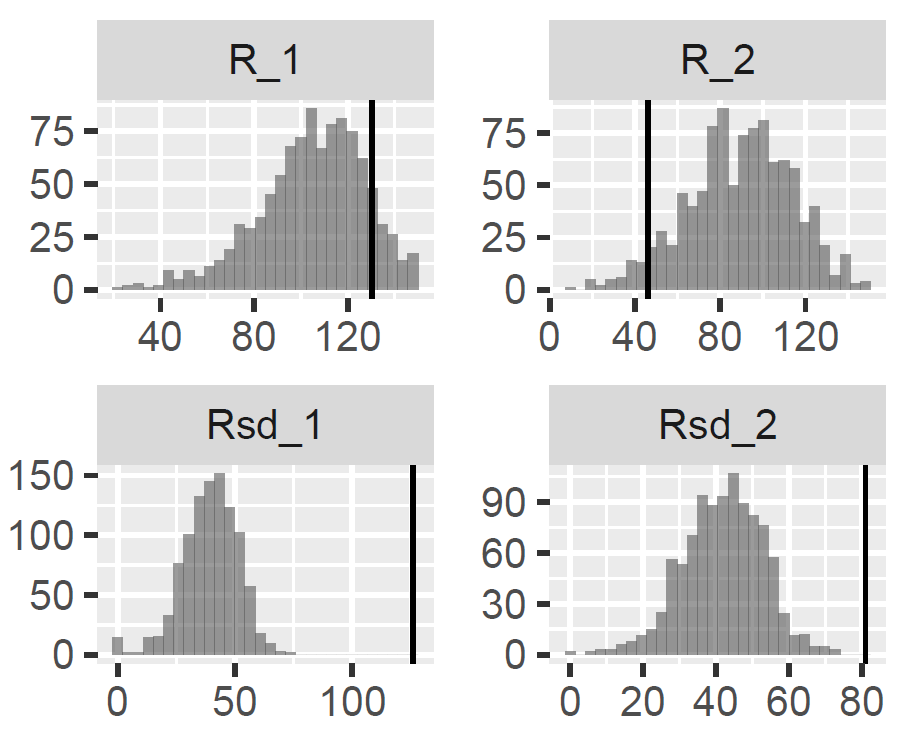

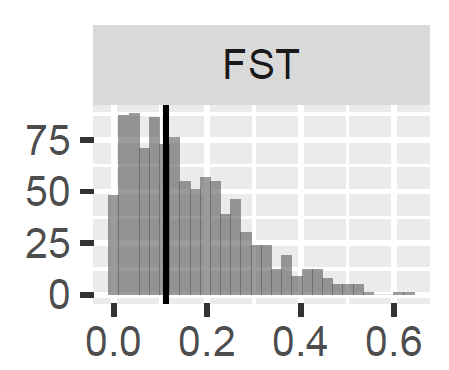

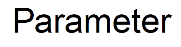

Supplement: Supplementary file 1 — Data S1 [file ECE3-13-e10500-s001.docx]
